# Interpretable Machine Learning–Derived Nomogram Model to Predict Population Health Literacy: Cross-Sectional Development and Validation Study

**DOI:** 10.2196/77654

**Published:** 2026-10-13

**Authors:** Xuehai Zhang, Haojie Jin, Zhiming Xie, Yan Li, Zishuo Huang, Chenkai Zhang, Yue Xu, Dingming Yao, Xiujing Hu, Yuyang Zhu, Xuebo Zhu, Chishuang Wang, Chun Chen

**Affiliations:** 1 Zhejiang Center for Disease Control and Prevention, Hangzhou, China; 2 School of Medical Humanities and Management, Wenzhou Medical University, Tongren Building 7B312, Wenzhou, 325035, China, 86 577 8669 95; 3 Cixi Biomedical Research Institute, Wenzhou Medical University, Ningbo; 4 School of Public Health, Wenzhou Medical University, Wenzhou, China; 5 Department of Psychology, University of California, Los Angeles, CA, United States; 6 Key Research Center of Philosophy and Social Sciences of Zhejiang Province, Institute of Medical Humanities, Wenzhou Medical University, Wenzhou, China

**Keywords:** health literacy, machine learning, nomogram, Shapley additive explanations, Zhejiang

## Abstract

**Background:**

Health literacy (HL) is a major factor associated with population health. However, no visualized predictive model has been developed to identify populations with low HL in a cost-effective manner.

**Objective:**

This study aims to identify key determinants of HL and develop a predictive nomogram model for population-level HL screening.

**Methods:**

This study analyzed cross-sectional data from 56,340 residents (aged 15‐69 y) in Zhejiang Province (2023) And used the Chinese Health Literacy Scale to measure HL. We used 3 complementary analytical approaches: logistic regression (LR) analysis to identify HL-associated factors, machine learning models (gradient boosting decision tree [GBDT], extreme gradient boosting [XGBoost], LR, and random forest [RF]) with Shapley additive explanations (SHAP) to quantify predictor importance, and nomogram development using 2023 data with external validation on 2024 data. In the nomogram, model 1 used all the predictive variables, while model 2 used the 5 core predictive variables determined through SHAP analysis. Model performance was evaluated using receiver operating characteristic curves, calibration plots, and decision curve analysis.

**Results:**

The nomogram models designed for predicting HL demonstrated robust stability, with good results in external validation. The area under the curve (AUC) values for both the test and validation sets of the machine learning models exceeded 0.75. At the 0.5 threshold, the sensitivities of the GBDT, XGBoost, LR, and RF test sets and validation sets were all >60%. The specificity of the test sets and validation sets of all 4 machine learning models was >75%. The positive predictive value (PPV) and negative predictive value (NPV) were both >60%. The AUC value of nomogram model 1 developed using the data from 2023 was 0.821, and the AUC value of the externally validated nomogram model 1 was 0.814. Similarly, nomogram model 2 had AUC values of 0.819 in the internal validation and 0.812 in the external validation. The PPV and NPV of both the internal validation model 1 and model 2 were all >60%. The integration of GBDT, XGBoost, LR, and RF models, along with the SHAP method, highlighted education level, age, annual household income, smoking, and occupation as the 5 most critical determinants.

**Conclusions:**

By using the GBDT, XGBoost, LR, RF, and nomogram models, a predictive model with substantial efficacy was effectively developed and validated for forecasting the HL levels of the population. This comprehensive model not only exhibits a commendable predictive capacity but also identifies the pivotal factors influencing HL. Such insights are crucial for shaping health policies and fostering healthier behaviors across the population.

## Introduction

Health literacy refers to the ability of accessing, understanding, and applying health information to promote health [1]. The concept of health literacy was first proposed by Simonds in his book *Health Education as Social Policy* in 1974 [2]. In recent years, with the popularization of health education and people’s increasing attention to health issues, research on health literacy has also received widespread attention [3]. Health literacy is a significant factor related to an individual’s health. Research indicates that individuals with lower health literacy often have a higher prevalence of chronic and infectious diseases as well as higher mortality rates. Additionally, there is a notable correlation between low health literacy and increased rates of disability [4-6]. For mental health, health literacy was negatively associated with depressive symptoms, and those with high health literacy were significantly less likely to have anxiety and sleep disorders [7,8]. In addition, previous studies have found that health literacy may increase engagement in social activities as well as enhance self-management abilities [9,10].

Over the past few decades, China has made great strides at improving the health literacy of the entire population, but disparities between regions and populations still exist. From 2022 to 2023, the proportion of people with health literacy in the economically developed eastern region saw a rise from 31.88% to 33.30%. In contrast, the health literacy level of residents in the less developed western regions in 2023 was only 24.44%, lower than the national average of 29.70%. In addition, the health literacy of the urban population is higher than in rural areas [11,12]. Meanwhile, there are large differences in health literacy levels between age groups, with youth and middle-aged individuals having higher levels of health literacy than older adults [13,14]. To address the imbalance in health literacy among regions and their populations and to raise the level of health literacy in the community, the government needs to pay more attention to populations with limited health literacy, formulate health policies in a targeted manner, and allocate resources to them.

Therefore, the key step to improving health literacy among the entire population is to identify populations with limited health literacy. Currently, the available methods for assessing health literacy are rather restricted, predominantly relying on extensive questionnaire-based assessments [15,16]. Typically, conducting a survey questionnaire involves substantial investments of human and material resources, necessitating comprehensive training for the personnel involved in administering the questionnaire, which can result in significantly elevated costs. In response to this issue, scholars have used machine learning methods to streamline the items in a functional health literacy survey questionnaire for Denmark [17]. In previous research, scholars have used machine learning methods to determine the relative importance of the primary factors associated with health literacy [18]. Additionally, researchers have conducted predictive assessments on whether residents and employees possess infectious disease health literacy using machine learning models based on the synthetic minority over-sampling technique (SMOTE) [19]. However, the factors influencing health literacy differ by region; therefore, the model might not apply in China. Studies have been conducted with the Chinese population using machine models and Shapley additive explanations (SHAP) analysis [20]. Furthermore, machine learning prediction models tend to be less intuitive and user-friendly in their application. Therefore, there is an urgent need for a low-cost, highly accurate, and more intuitive tool to predict the Chinese population’s health literacy.

Consequently, we used nomograms and machine learning to predict health literacy. Nomograms serve as a visualization tool that intuitively communicates model predictions, enhancing their comprehensibility. Furthermore, they can be developed into interactive web applications, enabling swift computation of an individual’s likelihood of possessing health literacy without requiring specialized knowledge or complex software [21,22]. Nomograms have found widespread application across various domains, including medical predictions for diverse diseases [23], energy sector forecasts for generator output [24], and environmental assessments of tropical soil suitability [25], among others.

To our knowledge, few studies have used nomograms to predict health literacy in the Chinese context. We used the interpretability and simplicity of nomograms as well as the effectiveness and robustness of machine learning algorithms to develop and evaluate predictive models for assessing population health literacy. The proposed predictive model holds the potential to significantly enhance holistic individual health management, facilitate optimal allocation of medical and educational resources, and inform the formulation of pertinent policies.

## Methods

### Data Sources

Data were derived from the 2023 Zhejiang Citizen Health Literacy and Tobacco Use Questionnaire administered by the Zhejiang Provincial Center for Disease Control and Prevention (March 2023-September 2023). The survey encompassed 56,340 respondents across 90 counties in 11 prefectural-level cities, collecting data on health literacy, tobacco use, and sociodemographic characteristics.

The study population comprised community-dwelling Chinese residents aged 15 to 69 years. We excluded individuals residing in institutional settings (military bases, hospitals, prisons, nursing homes, dormitories). The age range was selected to capture individuals who had completed compulsory education (≥15 y) and minimize potential cognitive impairment bias (<70 y).

This cross-sectional study was part of the 2023 China Health Literacy Survey, targeting noncollective Chinese residents aged 15 to 69 years in counties (cities and districts) across Zhejiang Province, excluding those living in military bases, hospitals, prisons, nursing homes, and dormitories. We excluded individuals younger than 15 years, as this age group typically has not completed compulsory education; we also excluded those older than 69 years, as this age group is more prone to cognitive impairment [26]. The external validation used data from 2024, which included a sample size of 56,863 individuals, also derived from the questionnaire survey on citizens' health literacy and tobacco use conducted by the Zhejiang Provincial Center for Disease Control and Prevention in Zhejiang Province [27]. During the data processing stage, no missing nor omitted entries were found, so there was no need for imputation of missing values. As a result, we obtained 56,340 complete and valid samples for the year 2023 and 56,863 complete and valid samples for the year 2024. The data acquisition rate was 100% [27].

### Outcome Variable: Health Literacy

The first part of the questionnaire assesses health literacy using the Chinese Health Literacy Scale developed by the Chinese Health Education Center using the Delphi method, comprising 50 items across 3 dimensions: knowledge and attitudes, behaviors and lifestyle, and health-related skills [28]. The overall Cronbach α for the scale was 0.95, and the Spearman-Brown coefficient was 0.94 [29].

The scale includes 3 question types: true/false (1 point for correct), single answer (1 point for choosing the correct one from multiple options), and multiple answers (2 points for selecting all correct answers from several options). If the health literacy score is ≥53, it implies that residents possess good health literacy (health literacy=1). Conversely, if the health literacy score is <53, it suggests that residents have limited health literacy (health literacy=0) [30].

### Predictors

We included a set of predictors based on previous studies. The set included sex (male or female), the continuous variable age (modeled as a continuous variable in all regression and machine learning analyses; the age brackets were quantile-based groupings for descriptive purposes only), ethnicity (Han or Minority), marital status (married or unmarried/divorced), education level (primary school and below, junior high school, senior high school or higher education including junior college, bachelor’s degrees, and postgraduate degrees) [31,32], occupation (technical/professional, student, manual, commercial, or unemployed/others), family size (1‐2, 3‐5, or >5), annual household income (0‐49,999, 50,000‐99,999, 100,000‐149,999, or ≥150,000), local registered residence (yes or no), current smoking (yes or no), presence of chronic diseases (yes or no), and self-rated health (very good, good, fair, bad, or very bad) [13,33-36]. The unemployment/other occupation group was set as the reference, and 4 dummy variables were established: technical/professional (Occupation_1), student (Occupation_2), manual (Occupation_3), and commercial (Occupation_4).

### Statistical Analysis

We used STATA 18.0, Scikit-Learn, and SHAP for gradient boosting decision tree (GBDT), extreme gradient boosting (XGBoost), logistic regression (LR), and random forest (RF) models in Python (version 3.8.10), and the “rmda” package for Nomogram in R (version 4.5.1) was used to perform statistical analyses. The nomogram was constructed based on LR. The 2 machine learning models, GBDT and XGBoost, excel at handling structured datasets and imbalanced datasets [37]. LR is a machine learning model mainly used for binary classification [38], while RF is a collection of decision trees that can reduce the adverse effects of overfitting [39]. In the 4 machine learning models, we randomly divided the data into a training set, validation set, and test set at an allocation ratio of 6:2:2. We also applied SMOTE to the training set. Meanwhile, we analyzed the performance indicators of the 4 models under the threshold of 0.1‐0.9 (Table S1 in Multimedia Appendix 1). Univariate and multivariable LR analyses were used to identify risk factors associated with health literacy.

GBDT is an ensemble learning algorithm that builds an ensemble of weak prediction models, typically decision trees, with the aim of improving the predictive accuracy of the model. GBDT is known for its ability to handle complex relationships and interactions between features, making it suitable for our dataset that has a large number of variables [40]. It is a widely used machine learning algorithm with high efficiency, accuracy, and interpretability. GBDT achieves state-of-the-art performance in many machine learning tasks such as multiclass classification, click prediction, and learning to sort [41].

XGBoost is an optimization of the gradient boosting framework that is designed to be highly efficient, flexible, and scalable. XGBoost differs from GBDT in its regularization approach, which helps prevent overfitting by adding a penalty term to the loss function for larger numbers of trees and deeper trees. This makes XGBoost particularly effective at handling large datasets with complex interactions while being efficient in terms of training time [42]. In addition, the advantages of XGBoost are that it can handle various types of data as well as missing values and outliers. It has the flexibility to choose different loss functions and weak predictive models, and it uses 10-fold cross-validation as a model evaluation strategy to avoid overfitting [43].

LR is the most commonly used simple machine learning model, mainly applied to binary classification problems. The core idea is to extend the linear regression model by using the sigmoid function to map the predicted values to the range of [0, 1]. LR has been widely applied in various fields of medicine, such as the prediction of cardiovascular diseases [44].

RF is an ensemble model composed of multiple decision trees. Each tree is grown randomly from the training set, and the final prediction is made by integrating the results of all the trees [45]. It can significantly reduce overfitting and increase prediction accuracy [46].

SHAP is a method for interpreting the output of any machine learning model. It is based on the concept of Shapley value in game theory and assigns a fair contribution score to each feature or feature value to indicate their effect on the model prediction. SHAP can not only help us to understand how the different features affect the model output and the magnitude of each feature’s contribution but also provide various visualizations of the global and local effects of features on the model [47].

A nomogram is a graphical representation that allows for the visualization of the relationship between multiple predictors and the outcome based on the results of a regression analysis. In our study, the data for 2023 were further divided into a development dataset and an internal validation dataset in a 7:3 ratio to create the nomogram model [48]. This division allowed us to develop the nomogram on the development dataset and validate its performance on the internal validation dataset, ensuring that the model’s predictive accuracy and generalizability were assessed [49]. A nomogram, as a visual model, can predict the probability of clinical outcomes and assist clinicians with making judgments [50].

Calibration curves were used to evaluate the performance of the nomogram model by comparing the predicted probabilities with the observed outcomes. This method provides a visual representation of the model’s calibration, with an ideal calibration curve being a diagonal line, indicating perfect agreement between predicted and observed probabilities [51]. Calibration curves are commonly used to evaluate models such as LR and Cox regression [52]. In this paper, we used it to assess the effectiveness of the nomogram model.

As shown in Table 1, to evaluate the performance of each machine learning model, we referred to and included the following indicators: area under the receiver operating characteristic (ROC) curve (AUC), sensitivity (equation 1), specificity (equation 2), accuracy (equation 3), positive predictive value (PPV; equation 6), negative predictive value (NPV; equation 7), and balanced accuracy (equation 5) [53,54]. Among them, balanced accuracy is the arithmetic mean of sensitivity and specificity [55]. Each machine learning model measured the probability that a resident had a high health literacy level. To further assess and interpret the predictive models, we calculated the net benefit of the nomogram model using decision curve analysis (DCA; equation 4). True positive (TP) and true negative (TN) indicate residents who were correctly identified as having a high level of health literacy or a low level of health literacy, respectively; false positive (FP) and false negative (FN) indicate residents who were inaccurately identified as having a high level of health literacy or a low level of health literacy, respectively. In binary classification, machine learning models predict the likelihood of individuals having health literacy. A threshold of 0.5 was used, classifying those with a probability of ≥0.5 as having a high level of health literacy and those with a probability <0.5 as not having a high level of health literacy.

**Table 1.** Equations for sensitivity, specificity, accuracy, net benefit, balanced accuracy, positive predictive value (PPV), and negative predictive value (NPV).

Equation number	Equation
Equation 1	Sensitivity=TP^a^/(TP+FN^b^)
Equation 2	Specificity=TN^c^/(TN+FP)^d^
Equation 3	Accuracy=(TP+TN)/(TP+TN+FN+FP)
Equation 4	Net benefit=(sensitivity×prevalence)–(1–specificity)*(1–prevalence)[P_t_^e^/(1-P_t_)]
Equation 5	Balanced accuracy=(sensitivity+specificity)/2
Equation 6	PPV=TP/(TP+FP)
Equation 7	NPV=TN/(TN+FN)

a TP: true positive.

b FN: false negative.

c TN: true negative.

d FP: false positive.

e P_t_: threshold probability.

### Ethical Considerations

#### Ethical Review Statement

The data analysis plan for this study was reviewed and approved by the Ethics Committee of the Zhejiang Provincial Center for Disease Control and Prevention of China (approval number: 2022–027‐01), and it complies with relevant laws and institutional regulations, including the Measures for Ethical Review of Life Science and Medical Research Involving Humans, the Personal Information Protection Law of the People’s Republic of China, and the Data Security Law of the People‘s Republic of China. All data were collected, stored, and analyzed in accordance with these requirements.

#### Informed Consent

The participants gave their informed consent during the original data collection. According to the relevant national laws and regulations as well as the institutional requirements, the ethics committee waived the need for a written informed consent request for this study.

#### Privacy and Confidentiality Protection

To protect the privacy of the participants, all collected data underwent de-identification processing and were only used in an anonymous form for analysis and reporting. The research data are stored in encrypted storage devices and are accessible only to authorized researchers.

#### Compensation

The participants of the questionnaire survey received an average gift worth ¥60 (US $8.94).

#### Protection of Images and Identifiable Information

No images that could identify the identities of the participants were used in this article or multimedia appendix.

## Results

As shown in Table 2, 56,340 participants in 2023 were included in this study. The number of respondents by gender did not differ significantly, with 26,357 (26,357/56,340, 46.78%) men and 29,983 (29,983/56,340, 53.22%) women, and there was not a significant difference in the mean health literacy scores by gender. Additionally, we conducted a demographic and health literacy score analysis on data from 56,863 participants in 2024 (Table S4 in Multimedia Appendix 1). Compared with 2023, the number of respondents in the survey was larger in 2024, and the average health literacy score was lower. There was no significant difference in the average health literacy score between men and women. In terms of population structure, there was little difference between 2023 and 2024.

**Table 2.** Demographic characteristics and average health literacy scores of the research population in the 2023 cross-sectional study in Zhejiang Province.

Predictors	Participants (N=56,340), n (%)	Health literacy score, mean (SD)
Entire sample	56,340 (100)	43.827 (13.446)
Age group (years)		
15‐33	8286 (14.71)	52.610 (8.646)
34‐41	7959 (14.13)	51.131 (9.814)
42‐49	8533 (15.15)	47.368 (11.852)
50‐54	7638 (13.56)	42.802 (12.857)
55‐59	8704 (15.45)	39.323 (13.134)
60‐64	7433 (13.19)	37.731 (13.215)
65‐69	7787 (13.82)	34.995 (12.925)
Gender		
Male	26,357 (46.78)	44.184 (13.110)
Female	29,983 (53.22)	43.513 (13.726)
Ethnicity		
Han	55,545 (98.59)	43.835 (13.445)
Other	795 (1.41)	43.281 (13.511)
Educational level		
Primary school and below	15,099 (26.80)	33.67 (12.66)
Junior high school	18,010 (31.97)	41.91 (12.25)
Senior high school	9824 (17.44)	48.91 (9.99)
Higher education	13,407 (23.80)	54.11 (7.64)
Marital status		
Single/widow/divorced	10,449 (18.55)	46.121 (13.315)
Married	45,891 (81.45)	43.304 (13.421)
Occupation		
Technical/professional (Occupation_1)	6708 (11.91)	52.707 (9.502)
Student (Occupation_2)	1816 (3.22)	52.685 (7.818)
Manual (Occupation_3)	28,231 (50.11)	39.041 (13.419)
Commercial (Occupation_4)	11,641 (20.66)	49.378 (10.887)
Unemployed/others	7944 (14.10)	43.176 (12.922)
Number of family members		
1‐2	18,969 (33.67)	40.777 (13.709)
3‐5	32,233 (57.21)	45.693 (12.918)
>5	5138 (9.12)	43.380 (13.614)
Annual household income (¥)		
0‐49,999	15,568 (27.63)	38.238 (13.920)
50,000‐99,999	13,549 (24.05)	42.340 (13.040)
100,000‐149,999	12,644 (22.44)	45.487 (12.435)
≥150,000	14,579 (25.88)	49.737 (11.266)
Local registered residence		
Yes	52,229 (92.7)	43.670 (13.494)
No	4111 (7.3)	45.823 (12.644)
Smoking		
No	46,090 (81.81)	44.150 (13.500)
Yes	10,250 (18.19)	42.374 (13.106)
Chronic disease		
No	40,702 (72.24)	45.488 (13.071)
Yes	15,638 (27.76)	39.502 (13.446)
Self-rated health		
Very good	18,337 (32.55)	44.253 (13.312)
Good	18,905 (33.56)	46.171 (12.789)
Fair	16,753 (29.74)	41.722 (13.573)
Bad	1851 (3.29)	37.830 (13.837)
Very bad	494 (0.88)	32.146 (13.390)

We also analyzed crude and adjusted odds ratios (ORs) for health literacy of the population using single and multiple variable binary LR analysis (Table 3). Although there was no significant difference in average health literacy scores between men and women (Table 2), after adjusting for confounding factors such as socioeconomic and demographic variables, the results showed that women were more likely to have lower health literacy (adjusted OR=0.902, 95% CI 0.860‐0.947), while no significant difference was observed between Han Chinese and ethnic minorities. Moreover, the health literacy level increased with education level. Those with junior high school, senior high school, and higher education had higher health literacy levels than those with a primary school education and below, and the highest levels of health literacy were found among those with higher education (adjusted OR=14.604, 95% CI 13.256‐16.089). Marital status also affected health literacy, although Table 2 shows that the average health literacy score of those with a partner was lower than that of single, divorced, or widowed individuals after adjusting for confounding factors and those who had partners (adjusted OR=1.253, 95% CI 1.176‐1.336) had higher health literacy than those who were single, divorced, or widowed. Furthermore, occupation was related to health literacy; the health literacy levels of technical and professional workers were higher than those of people in other occupations. Table 2 shows that the average health literacy score of those with 3‐5 family members was higher. However, after adjusting for confounding factors, the number of family members was not a significant independent predictor of health literacy level. Additionally, annual household income and household registration were associated with the health literacy, although Table 2 shows that the average health literacy score of nonlocal registered residents was higher than that of local registered residents after adjusting for confounding factors and those who had a higher income and local registration had higher health literacy levels than those who had lower income and nonlocal registration (adjusted OR=0.654, 95% CI 0.605‐0.706). Finally, health behaviors and conditions also influenced health literacy levels, and those who smoked (adjusted OR=0.814, 95% CI 0.765‐0.866) and had chronic diseases (adjusted OR=0.931, 95% CI 0.880‐0.986) had lower health literacy levels than those who did not smoke and did not have chronic diseases, respectively. Self-rated health was also correlated with health literacy levels, and those who rated their health as good had higher health literacy levels than those who rated their health as bad.

**Table 3.** The crude odds ratios (ORs) and adjusted ORs obtained through logistic regression analysis of the factors related to residents’ health literacy in Zhejiang province in 2023.

Predictors	Crude OR (95% CI)	*P* value	Adjusted OR (95% CI)	*P* value
Age (years)	0.934 (0.932-0.935)	<.001	0.970 (0.967-0.972)	<.001
Gender				
Male	Reference	—^a^	Reference	—
Female	0.974 (0.940-1.009)	.14	0.902 (0.860-0.947)	<.001
Ethnicity				
Han	Reference	—	Reference	—
Other	0.943 (0.811-1.096)	.45	1.043 (0.873-1.245)	.64
Educational level				
Primary school and below	Reference	—	Reference	—
Junior high school	4.390 (4.070-4.734)	<.001	3.338 (3.087-3.609)	<.001
Senior high school	11.968 (11.072-12.937)	<.001	6.713 (6.150-7.328)	<.001
Higher education	35.346 (32.751-38.148)	<.001	14.604 (13.256-16.089)	<.001
Marital status				
Single/widow/divorced	Reference	—	Reference	—
Married	0.612 (0.585-0.639)	<.001	1.253 (1.176-1.336)	<.001
Occupation				
Technical/professional (Occupation_1)	4.284 (4.061-4.518)	<.001	1.317 (1.214-1.429)	<.001
Student (Occupation_2)	3.301 (3.000-3.634)	<.001	0.921 (0.808-1.050)	.22
Manual (Occupation_3)	0.241 (0.232-0.250)	<.001	1.100 (1.029-1.176)	.005
Commercial (Occupation_4)	2.520 (2.417-2.628)	<.001	1.083 (1.008-1.163)	.03
Unemployed/others	Reference	—	Reference	—
Number of family members				
1‐2	Reference	—	Reference	—
3‐5	1.970 (1.893-2.051)	<.001	1.052 (1.000-1.107)	.051
>5	1.470 (1.374-1.573)	<.001	0.994 (0.915-1.080)	.88
Annual household income (¥)				
0‐49,999	Reference	—	Reference	—
50,000‐99,999	1.581 (1.495-1.672)	<.001	1.306 (1.226-1.391)	<.001
100,000‐149,999	2.520 (2.386-2.662)	<.001	1.324 (1.242-1.411)	<.001
≥150,000	5.009 (4.755-5.278)	<.001	1.681 (1.576-1.792)	<.001
Local registered residence				
Yes	Reference	—	Reference	—
No	1.285 (1.204-1.372)	<.001	0.654 (0.605-0.706)	<.001
Smoking				
No	Reference	—	Reference	—
Yes	0.703 (0.670-0.737)	<.001	0.814 (0.765-0.866)	<.001
Chronic disease				
No	Reference	—	Reference	—
Yes	0.392 (0.375-0.410)	<.001	0.931 (0.880-0.986)	.01
Self-rated health				
Very good	Reference	—	Reference	—
Good	1.296 (1.242-1.351)	<.001	1.163 (1.107-1.223)	<.001
Fair	0.689 (0.658-0.722)	<.001	1.009 (0.954-1.067)	.75
Bad	0.401 (0.354-0.454)	<.001	0.938 (0.812-1.085)	.39
Very bad	0.149 (0.105-0.210)	<.001	0.573 (0.397-0.827)	.003

a Not applicable.

Table 4 shows the performance in terms of AUC of the GBDT, XGBoost, LR, and RF models in the test set (GBDT: 0.810, 95% CI 0.801‐0.819; XGBoost: 0.795, 95% CI 0.786‐0.805; LR: 0.814, 95% CI 0.805‐0.823; RF: 0.817, 95% CI 0.808‐0.826) and in the validation set (GBDT: 0.807, 95% CI 0.798‐0.816; XGBoost: 0.789, 95% CI 0.780‐0.799; LR: 0.809, 95% CI 0.799‐0.818; RF: 0.813, 95% CI 0.804‐0.822; Figure 1). The AUC values of the 4 models in the test set and validation set were all >0.75, and the results in the test set and validation set were the same, which indicates that these 4 models are robust models for distinguishing residents with health literacy from those without. Meanwhile, at the 0.5 threshold, the specificity of the test set and validation set for the GBDT, XGBoost, LR, and RF models was >75%, and the LR model had the highest sensitivity in both the test set and validation set. Moreover, the PPV and NPV were both >60%. The performance indicators of the 4 models in the validation sets, which were at the 0.1‐0.9 threshold, showed that the AUC value of the RF model was the highest (0.813), and its performance was slightly better than that of the other models. As the threshold increased, the sensitivity and NPV of each model gradually decreased, while the specificity and PPV gradually increased (Table S1 in Multimedia Appendix 1). Since the AUC value of the RF validation set was the highest, the 2024 data were brought into RF for external validation. The AUC value of the 2024 validation set was 0.815, and various performance indicators under the 0.1‐0.9 threshold were calculated. As the threshold increased, sensitivity decreased while specificity increased (Table S2 in Multimedia Appendix 1).

**Table 4.** The 4 machine learning models (gradient boosted decision tree [GBDT], extreme gradient boosting [XGBoost], logistic regression [LR], and random forest [RF]) constructed based on the 2023 data were tested at the 0.5 threshold for various performance indicators of the test set and validation set.

Model	AUC^a^ (95% CI)	TN^b^	TP^c^	FN^d^	FP^e^	Sensitivity, (95% CI)	Specificity (95% CI)	Accuracy (95% CI)	Balanced accuracy (95% CI)	PPV^f^ (95% CI)	NPV^g^ (95% CI)
Test set											
GBDT	0.810 (0.801-0.819)	6167	2440	1325	1336	0.648 (0.633-0.663)	0.822 (0.813-0.831)	0.764 (0.756-0.772)	0.735 (0.726-0.744)	0.646 (0.631-0.661)	0.823 (0.815-0.832)
XGBoost	0.795 (0.78-0.805)	6047	2435	1330	1456	0.647 (0.631-0.662)	0.806 (0.797-0.815)	0.753 (0.745-0.761)	0.726 (0.717-0.735)	0.626 (0.611-0.641)	0.820 (0.811-0.828)
LR	0.814 (0.805-0.823)	5916	2645	1120	1587	0.703 (0.688-0.717)	0.788 (0.779-0.798)	0.760 (0.752-0.768)	0.746 (0.737-0.754)	0.625 (0.610-0.640)	0.841 (0.832-0.849)
RF	0.817 (0.808-0.826)	5971	2636	1129	1532	0.700 (0.685-0.715)	0.796 (0.787-0.805)	0.764 (0.756-0.772)	0.748 (0.739-0.757)	0.632 (0.618-0.647)	0.841 (0.832-0.849)
Validation											
GBDT	0.807 (0.798- 0.816)	6248	2310	1333	1377	0.634 (0.618-0.650)	0.819 (0.811-0.828)	0.759 (0.752-0.767)	0.727 (0.718-0.736)	0.627 (0.611-0.642)	0.824 (0.816-0.833)
XGBoost	0.789 (0.780-0.799)	6133	2291	1352	1492	0.629 (0.613-0.645)	0.804 (0.795-0.813)	0.748 (0.740-0.756)	0.717 (0.708-0.726)	0.606 (0.590-0.621)	0.819 (0.811-0.828)
LR	0.809 (0.799-0.818)	6030	2476	1167	1595	0.680 (0.665-0.695)	0.791 (0.782-0.800)	0.755 (0.747-0.763)	0.735 (0.726-0.744)	0.608 (0.593-0.623)	0.838 (0.829-0.846)
RF	0.813 (0.804-0.822)	6063	2475	1168	1562	0.679 (0.664-0.695)	0.795 (0.786-0.804)	0.758 (0.750-0.766)	0.737 (0.728-0.746)	0.613 (0.598-0.628)	0.838 (0.830-0.847)

a AUC: area under the curve.

b TN: true negative.

c TP: true positive.

d FN: false negative.

e FP: false positive.

f PPV: positive predictive value.

g NPV: negative predictive value.

**Figure 1.**
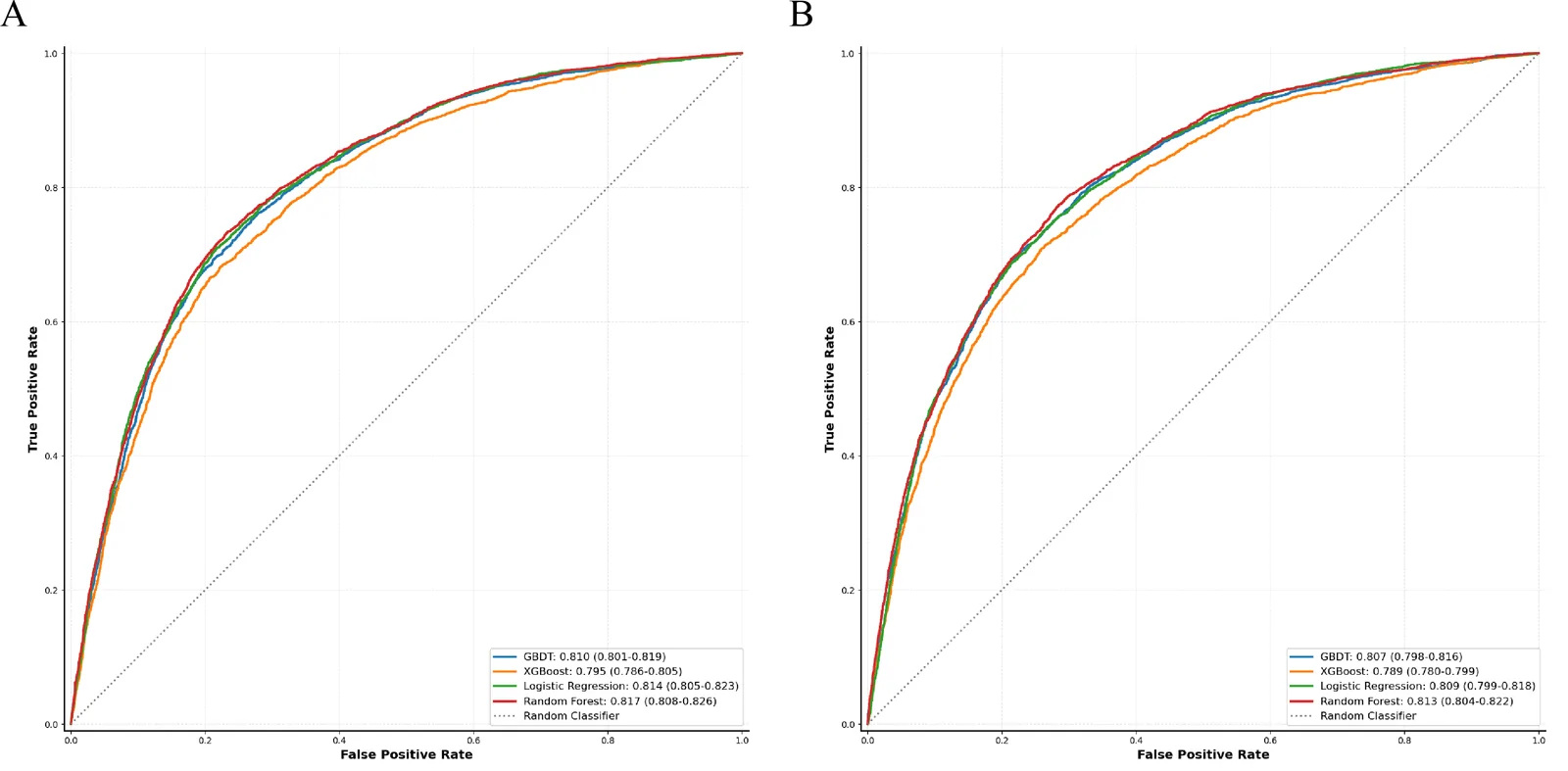
Comparison chart of receiver operating characteristic (ROC) curves for the (A) test set and (B) validation set of the 4 machine learning models (gradient boosting decision tree [GBDT], extreme gradient boosting [XGBoost], logistic regression, and random forest [RF]) constructed based on the 2023 data.

In nomogram model 1, we selected all the variables involved in LR analysis, including gender, age, ethnicity, marital status, education level, family size, annual household income, local registered residence, smoking, chronic conditions, self-rated health, and 4 dummy variables representing occupation (Figure 2). By analyzing the training set of GBDT, XGBoost, LR, and RF models using the SHAP method, we found that, based on the average rankings of the 15 variables in these 4 models, the top 5 most important features for predicting health literacy were education level, age, annual household income, smoking, and manual occupation (Occupation_3; Figure 3; Table 5). Therefore, we included these 5 variables and 3 additional dummy variables for occupation in nomogram model 2 for analysis (Figure 2).

**Figure 2.**
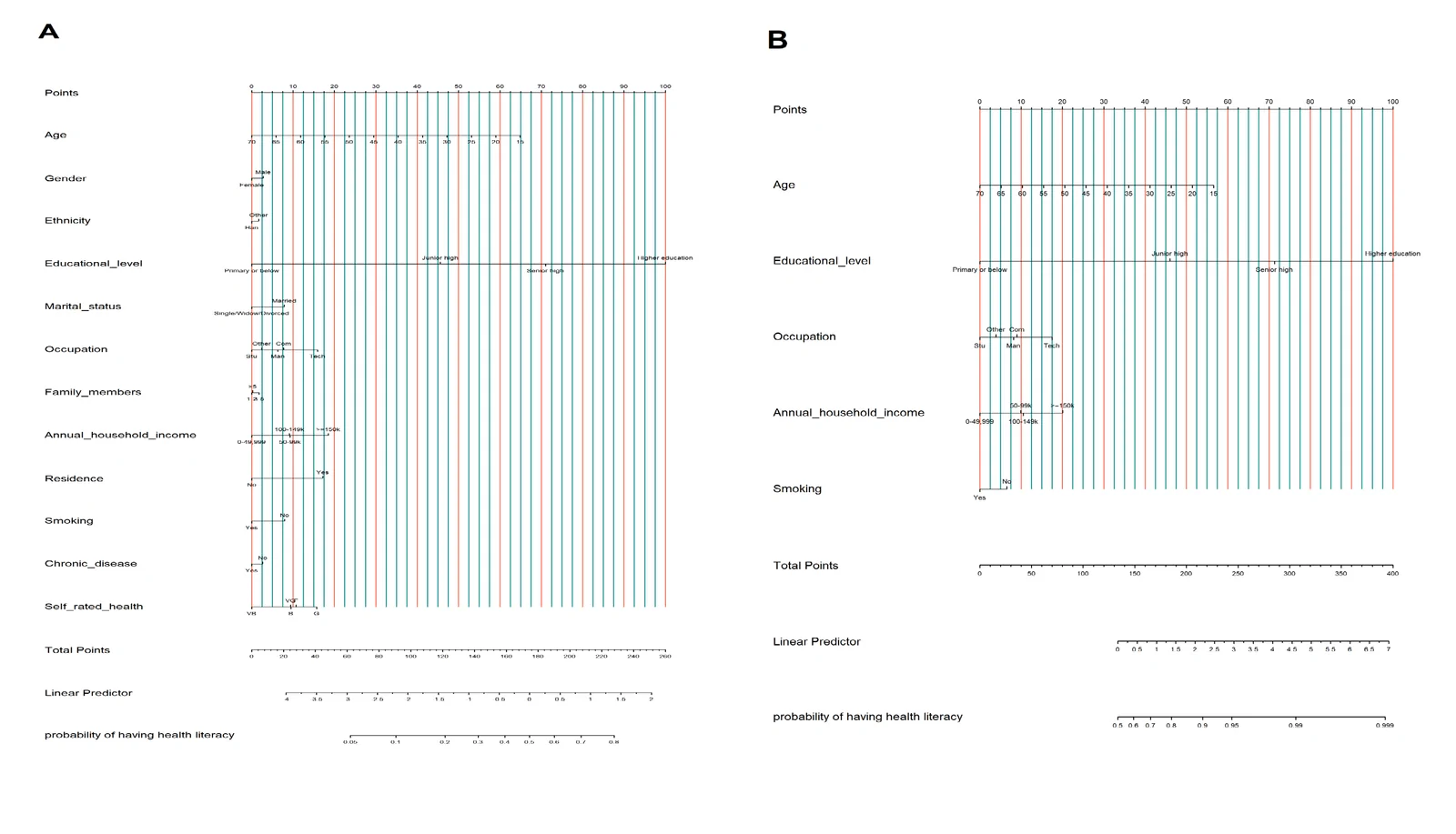
Nomogram models used to visually present the prediction of individual health literacy: (A) model 1 adjusted for gender, age, ethnicity, marital status, education level, occupation, number of family members, annual household income, local registered residence, smoking, chronic conditions, and self-rated health and (B) model 2 adjusted for educational level, age, annual household income, occupation, and smoking.

**Figure 3.**
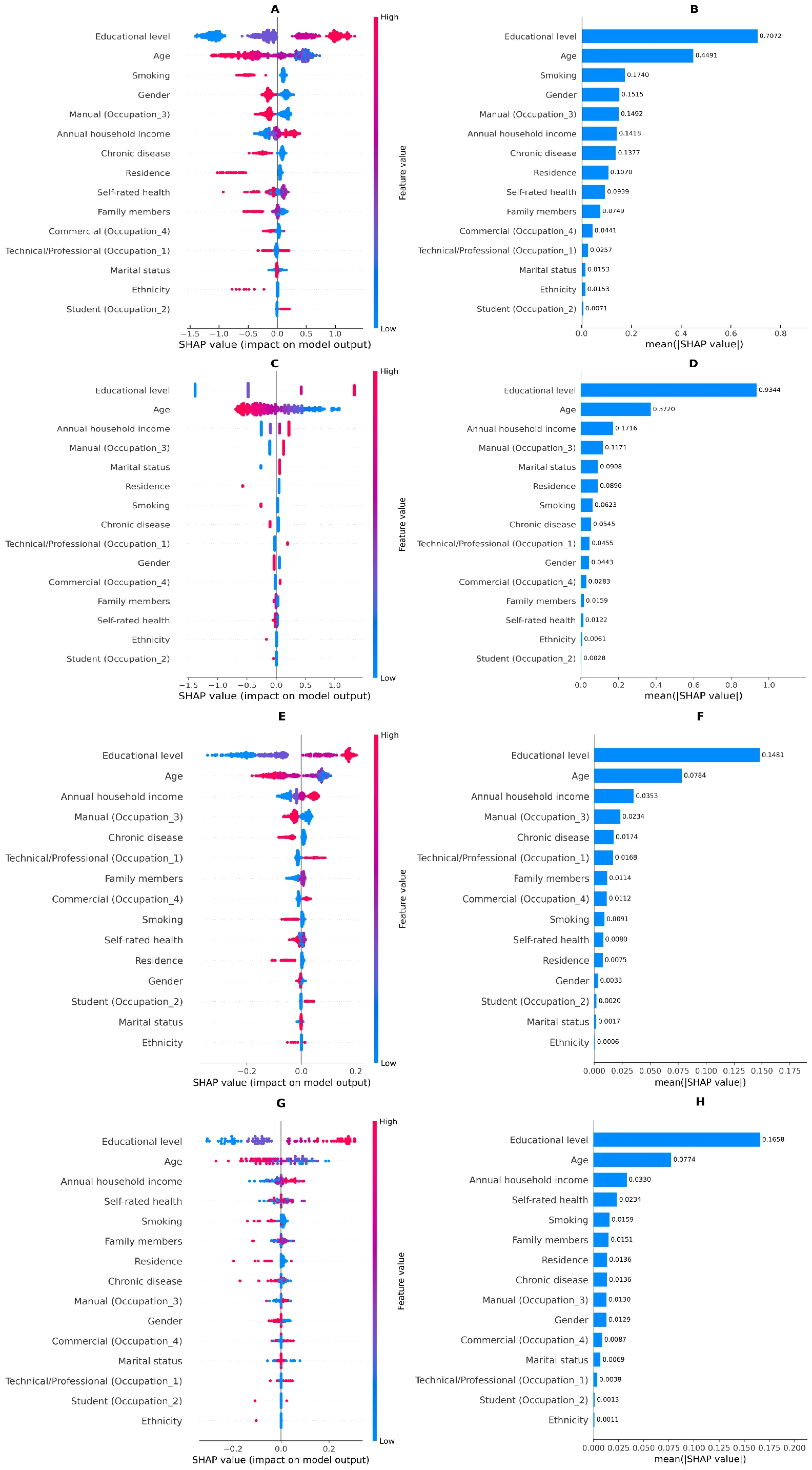
The Shapley additive explanations (SHAP) analysis explaining the influence of each feature on the predictions in the 4 machine learning models (gradient boosting decision tree [GBDT], extreme gradient boosting [XGBoost], logistic regression [LR], and random forest [RF]) constructed using the 2023 data; the larger the SHAP or mean SHAP value, the greater the influence, and a higher ranking on the y axis indicates a greater impact of the feature on the model output: (A) SHAP summary plot for GBDT, (B) feature importance for GBDT, (C) SHAP summary plot for LR, (D) feature importance for LR, (E) SHAP summary plot for RF, (F) feature importance for RF, (G) SHAP summary plot for XGBoost, (H) feature importance for XGBoost.

**Table 5.** Rankings of predictors in the Shapley additive explanations (SHAP) analysis of the 4 machine learning models as well as the final average ranking.

Predictors	SHAP analysis ranking of GBDT^a^	SHAP analysis ranking of XGBoost^b^	SHAP analysis ranking of LR^c^	SHAP analysis ranking of RF^d^	Average ranking
Age	2	2	2	2	2
Educational level	1	1	1	1	1
Annual household income	6	3	3	3	3.75
Smoking	3	5	7	9	6
Technical or professional (Occupation_1)	12	13	9	6	10
Student (Occupation_2)	15	14	15	13	14.25
Manual (Occupation_3)	5	9	4	4	5.5
Commercial (Occupation_4)	11	11	11	8	10.25
Number of family members	10	6	12	7	8.75
Gender	4	10	10	12	9
Ethnicity	14	15	14	15	14.5
Marital status	13	12	5	14	11
Residence	8	7	6	11	8
Chronic disease	7	8	8	5	7
Self-rated health	9	4	13	10	9

a GBDT: gradient boosting decision tree.

b XGBoost: extreme gradient boosting.

c LR: logistic regression.

d RF: random forest.

In nomogram models 1 and 2, the calibration was assessed on the development set (70% of the 2023 data) using bootstrap optimism correction. The calibration plots (Figure 4) display both the apparent and bias-corrected curves, both of which closely follow the ideal diagonal line (absolute error 0.003 for both models), confirming excellent agreement between predicted and observed probabilities. The ROC graphs for model 1 and model 2 of the internal validation show AUC values of 0.821 and 0.819 (Figure 5), respectively, and the ROC graphs for model 1 and model 2 of the external validation show AUC values of 0.814 and 0.812, respectively (Figure 6). The AUC values of the 2 models in the internal and external validation were >0.75. Furthermore, the performance indicators of the internal validation (30% of the 2023 data) models 1 and model 2 on the test sets within the threshold range of 0.3 to 0.7 show that, as the threshold increases, specificity increases while sensitivity decreases and PPV and the NPV were both >60% (Table 6). The performance indicators of the test sets for external validation models 1 and 2 under the threshold range of 0.1 to 0.9 showed that, as the threshold increased, specificity increased while sensitivity decreased (Table S3 in Multimedia Appendix 1).

**Figure 4.**
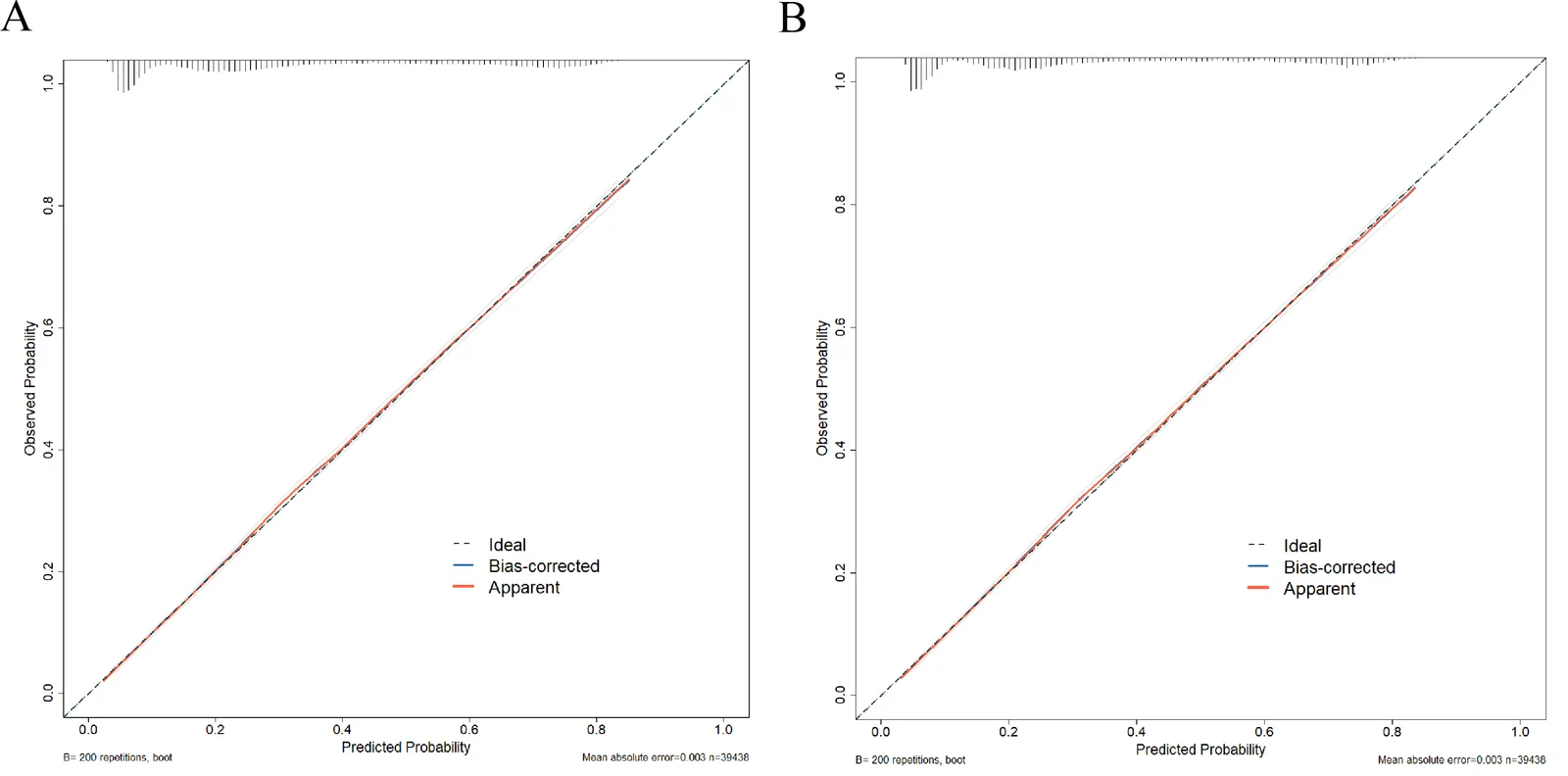
The calibration curves of nomogram (A) model 1 and (B) model 2 constructed based on the data from 2023.

**Figure 5.**
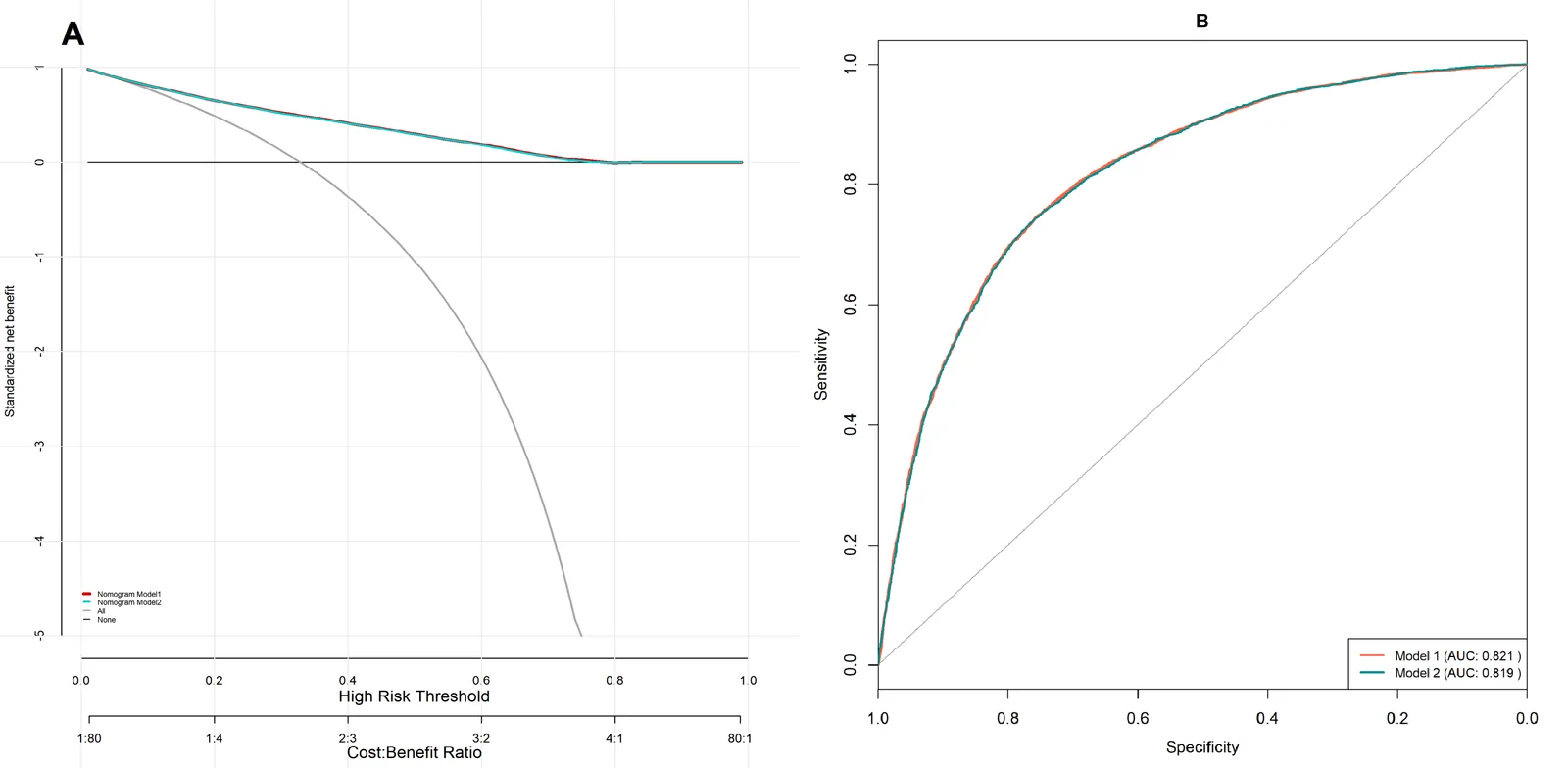
(A) Decision curve analysis (DCA) and (B) receiver operating characteristic (ROC) curve for internally validated nomogram models, developed and tested on the 2023 Zhejiang dataset. AUC: area under the curve.

**Figure 6.**
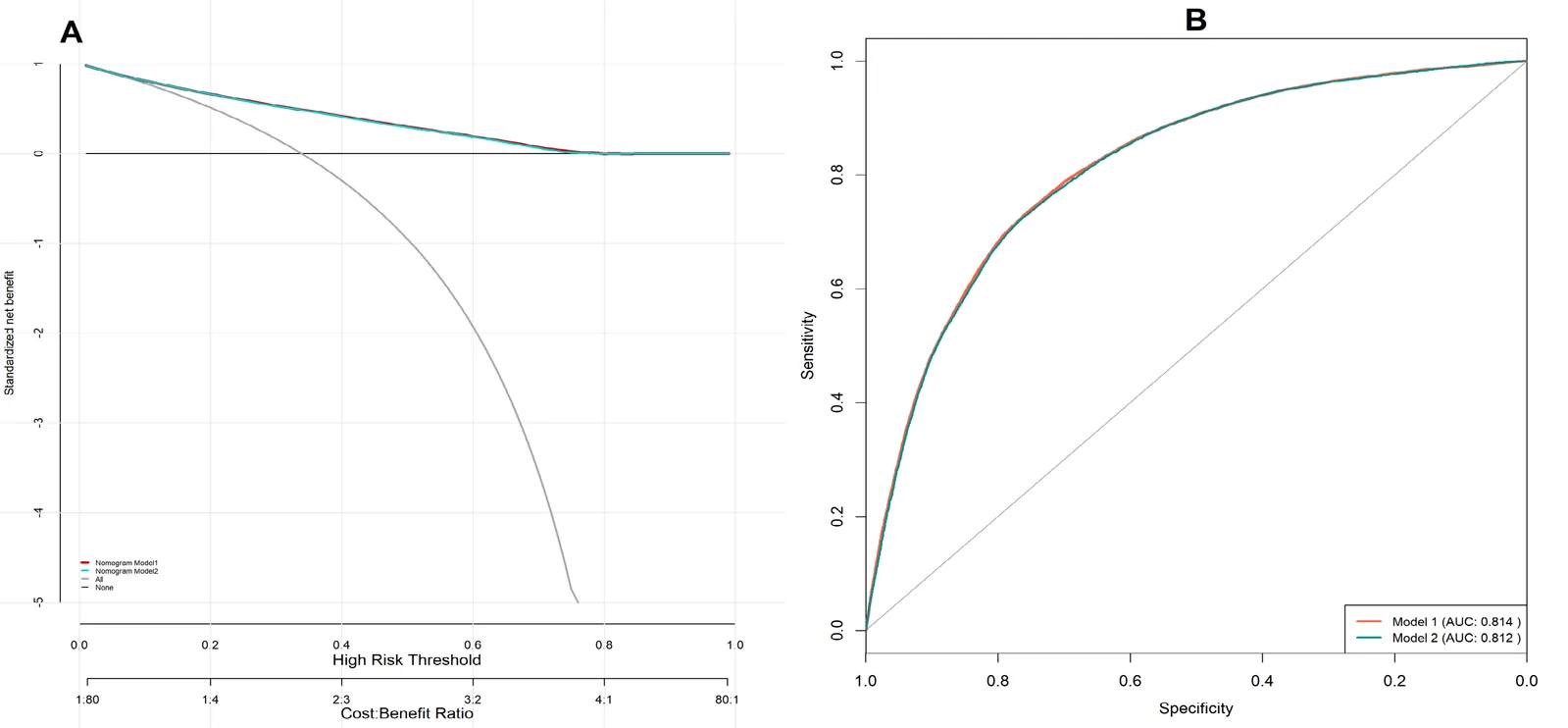
(A) Decision curve analysis (DCA) and (B) receiver operating characteristic (ROC) curve for the external validation of nomogram models 1 and 2 (developed based on 2023 data) on the 2024 dataset. AUC: area under the curve.

**Table 6.** Performance indicators of the internal validation models 1 and 2 under the threshold range of 0.3 to 0.7 in the internal validation set developed using 2023 data from Zhejiang.

Model and threshold	TN^a^	TP^b^	FN^c^	FP^d^	Sensitivity (95% CI)	Specificity (95% CI)	Accuracy (95% CI)	Balanced accuracy (95% CI)	PPV (95% CI)	NPV (95% CI)	AUC (95% CI)
Model 1											0.821 (0.814-0.827)
0.3	8944	3888	1662	2408	0.701 (0.688-0.713)	0.788 (0.780-0.795)	0.759 (0.753-0.766)	0.744 (0.737-0.751)	0.618 (0.606-0.630)	0.843 (0.836-0.850)	
0.4	9448	3547	2003	1904	0.639 (0.626-0.652)	0.832 (0.825-0.839)	0.769 (0.762-0.775)	0.736 (0.728-0.743)	0.651 (0.638-0.663)	0.825 (0.818-0.832)	
0.5	9801	3199	2351	1551	0.576 (0.563-0.589)	0.863 (0.857-0.870)	0.769 (0.763-0.775)	0.720 (0.713-0.727)	0.673 (0.660-0.687)	0.807 (0.800-0.814)	
0.6	10,103	2840	2710	1249	0.512 (0.499-0.525)	0.890 (0.884-0.896)	0.766 (0.759-0.772)	0.701 (0.694-0.708)	0.695 (0.680-0.709)	0.789 (0.781-0.796)	
0.7	10,375	2468	3082	977	0.445 (0.432-0.458)	0.914 (0.909-0.919)	0.760 (0.753-0.766)	0.679 (0.672-0.686)	0.716 (0.701-0.731)	0.771 (0.764-0.778)	
Model 2											0.819 (0.813-0.826)
0.3	9252	3703	1847	2100	0.667 (0.655-0.680)	0.815 (0.808-0.822)	0.766 (0.760-0.773)	0.741 (0.734-0.748)	0.638 (0.626-0.650)	0.834 (0.827-0.841)	
0.4	9496	3513	2037	1856	0.633 (0.620-0.646)	0.837 (0.830-0.843)	0.770 (0.763-0.776)	0.735 (0.728-0.742)	0.654 (0.642-0.667)	0.823 (0.816-0.830)	
0.5	9722	3288	2262	1630	0.592 (0.580-0.605)	0.856 (0.850-0.863)	0.770 (0.763-0.776)	0.724 (0.717-0.732)	0.669 (0.655-0.682)	0.811 (0.804-0.818)	
0.6	9909	3085	2465	1443	0.556 (0.543-0.569)	0.873 (0.867-0.879)	0.769 (0.762-0.775)	0.714 (0.707-0.722)	0.681 (0.668-0.695)	0.801 (0.794-0.808)	
0.7	10,099	2845	2705	1253	0.513 (0.499-0.526)	0.890 (0.884-0.895)	0.766 (0.759-0.772)	0.701 (0.694-0.708)	0.694 (0.680-0.708)	0.789 (0.782-0.796)	

a TN: true negative.

b TP: true positive.

c FN: false negative.

d FP: false positive.

Further evaluation through DCA indicated that, within the threshold range examined, the net benefits of the GBDT, LR, and RF models were consistently higher than those of the XGBoost model (Figure 7). All 4 models showed broadly comparable discrimination (AUC range 0.789‐0.813), and the DCA results suggested a potential advantage in net benefit for GBDT, LR, and RF under the evaluated probability thresholds. In the 2 DCA plots for the internally validated and externally validated nomogram models, the net gains for models 1 and 2 were very close to each other, suggesting that model 1 (incorporating 12 individual predictors, with occupation as a categorical predictor represented by 4 dummy variables) and model 2 (incorporating 5 core predictors [education level, age, annual household income, occupation, and smoking], with occupation as a categorical predictor represented by 4 dummy variables) performed similarly, as do the 2 models in external validation.

**Figure 7.**
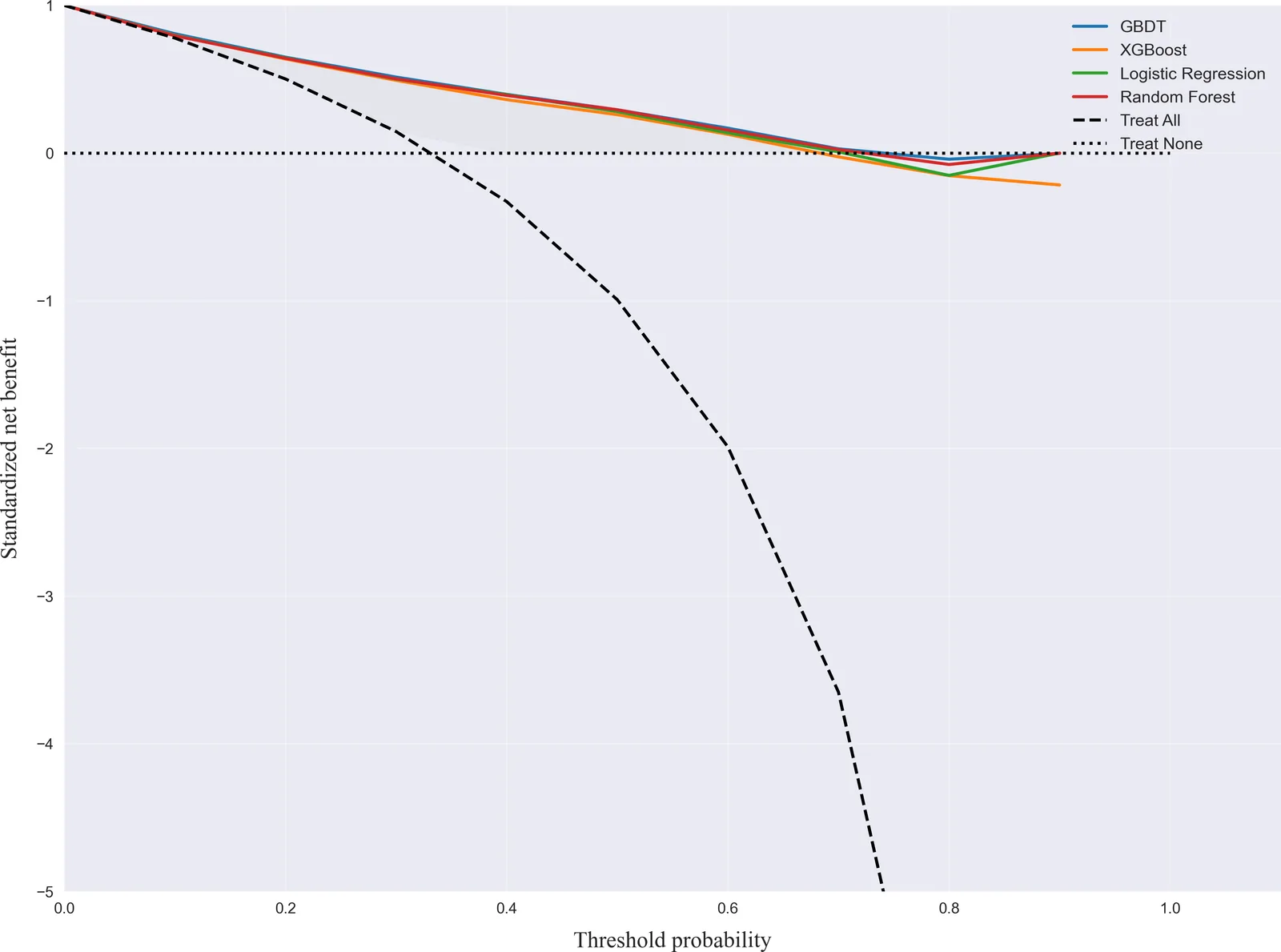
The decision curve analysis of the 4 machine learning models (gradient boosting decision tree [GBDT], extreme gradient boosting [XGBoost], logistic regression [LR], and random forest [RF]) for health literacy levels constructed based on the 2023 data.

## Discussion

### Principal Findings

To our best knowledge, this study conducted the first comprehensive approach combining nomograms and machine learning models to predict population health literacy, developed and externally validated using large-scale survey data. The models demonstrated robust performance across both development and external validation cohorts, establishing their utility for population-level health literacy prediction. The nomogram was constructed based on LR [56]. The AUC values of this nomogram were 0.821 in the internal validation model 1 and 0.814 in the external validation model 1, indicating its excellent predictive performance. Moreover, this nomogram is simple and easy to understand and has high interpretability in clinical practice.

In this study, the GBDT, XGBoost, LR, and RF models achieved AUC values exceeding 0.75 in both test and validation sets, indicating strong discriminatory power and low false prediction rates. To enhance interpretability and practicality, we constructed and validated 2 nomogram models. Both internally validated nomogram models and externally validated nomogram models exhibited diagnostic accuracy, as evidenced by their AUC values, and their calibration curves demonstrated good concordance between predicted and actual probabilities, signifying high precision at distinguishing individuals with differing levels of health literacy. SHAP analysis of the GBDT, XGBoost, LR, and RF models revealed 5 critical predictors for nomogram model 2. Despite varying variable compositions, models 1 and 2 exhibited strikingly similar performance, substantiating the viability of reducing the predictor set. In previous studies, nomograms combined with machine learning have been used to develop risk prediction models for cerebral infarction, eHealth literacy, and thymoma [57-59]. In conclusion, the results of machine learning and nomogram to predict diseases or other risks supported the feasibility and validity of the study hypothesis.

### Comparison With Prior Work and Influencing Factors

In this study, we further analyzed the performance indicators of the GBDT, XGBoost, LR, and RF models on the test set and validation set at the 0.5 threshold (Table 4) as well as the performance indicators of the 4 machine learning models on the validation set at the 0.1‐0.9 threshold (Table S1 in Multimedia Appendix 1). The health literacy prediction model demonstrated high efficacy for health literacy prediction, but at thresholds of 0.1 and 0.9, there were situations where the model’s sensitivity or specificity was low. Therefore, in practical applications, we should select an appropriate threshold to balance sensitivity and specificity, thereby achieving high accuracy and cost-effectiveness. As shown in Figure 7, when the threshold was 0.5, the LR model had a high net benefit and balanced sensitivity and specificity. The models constructed in this study contained 12 variables (with occupation as a categorical predictor represented by 4 dummy variables), which were more comprehensive than short health literacy screening tools such as the HL-SF3 [60] and HL-SF6 [61]. In the context of accelerating the construction of regional information health platforms and the digitization of health information in China [62], this model conducted population-based health literacy screening and monitoring based on big data. By integrating data from regional health information systems, this model can assess health literacy at the public health level, helping to identify health literacy gaps among different groups and regions. Moreover, the AUC values of model 1 and model 2 in the internal and external validations were both >0.75, indicating good predictive efficacy and the ability to provide a basis for policy formulation, resource allocation, and the implementation of targeted intervention measures, thereby improving overall health literacy levels.

To investigate the risk factors associated with health literacy, this study analyzed and integrated the findings from LR analysis and SHAP models. Overall education, age, occupation, household income, smoking, and self-rated health emerged as significant predictors related to health literacy. Studies have noted that education, age, and economic status are associated with health literacy [18,63]. Essentially, there was a strong positive relationship between educational attainment and health literacy, with individuals with higher education often having greater access to comprehensive health education and information. These individuals demonstrate enhanced abilities in understanding and processing such data, which can lead to a more effective grasp of medical advice, disease prevention measures, and the importance of health management [64]. Age has been identified as a key variable in health literacy, with a Beijing-based survey indicating higher health literacy among individuals aged 25 to 44 years [14]. Aging may enable the accumulation of health-related knowledge and skills through life experiences, but it can also be associated with cognitive decline, potentially affecting older adults’ ability to absorb and use health information [65]. Younger generations, on the other hand, may benefit from modern health education systems to gain access to updated health concepts and technologies [66]. Additionally, occupational disparities can influence health literacy. Professionals in technical or specialized fields often engage in specific training and continuous learning, which may include relevant health and safety knowledge and skill development [67]. For example, medical practitioners and educators, who benefit from advanced education, may also have access to favorable working environments and benefits such as health insurance and fitness facilities, factors that can contribute to their health literacy [68]. The association between household income and health literacy is well-documented. Individuals with higher economic status often have access to better educational resources and a wider range of in-depth health guidance, which are critical to enhancing their health literacy levels. Individuals who rate their health status positively may be more likely to actively monitor and manage their well-being, seeking out and applying health-related knowledge, which can result in higher levels of health literacy [13,69,70]. At the same time, there are studies that have shown that smoking habits are related to health literacy. Compared with smokers, nonsmokers have a higher level of health literacy [71]. Self-rated health is also related to health literacy [72]. Health literacy is defined as an individual’s ability to acquire, understand, and use health information and services and make appropriate health decisions [73]. Self-rated health is defined as an individual’s perception of their own health status [74]. Studies have shown that people who rate their own health as good have higher health literacy [72], while those who rate their health as average or poor have insufficient health literacy [75]. Meanwhile, the results of the LR and SHAP analyses showed that factors such as ethnicity were not significantly correlated with health literacy. Another cross-sectional study from Malaysia demonstrated that ethnicity was not significantly correlated with the level of health literacy [76].

### Implications and Future Applications

In this study, the health literacy prediction model exhibited commendable performance, highlighting its substantial potential for effective implementation and wide-ranging practical application in addressing health literacy challenges. At the individual level, individuals predicted to have high health literacy can act as catalysts, not only imparting health knowledge and skills to their peers but also extending these teachings to those around them, including older adults, thereby enhancing their health literacy. As health literacy improves, it can boost work productivity [77], heighten health awareness, facilitate better self-management, and elevate overall quality of life. On a broader societal canvas, the health literacy prediction model can seamlessly interface with health big data systems in the future, allowing for a systematic and exhaustive assessment of health literacy rates across different population groups and regions [78]. This approach stands to reduce the reliance on large-scale questionnaire surveys, thereby saving costs and resources. This capacity facilitates targeted interventions and the strategic roll out of health education programs in populations or regions with low health literacy scores, progressively closing health literacy gaps and promoting a more equitable distribution of health care and education resources. At the national level, leveraging the health literacy prediction model provides policymakers with a unique vantage point to foresee the contours of the country’s health literacy landscape. It can direct strategic fund allocation to health literacy–deficient areas, concurrently orchestrating the refinement of health strategies and bolstering investments in cultivating a skilled workforce of health care technology professionals. At the same time, the reliability of the model can be verified through national medical data, and it can be incorporated into national primary health care work to monitor the health literacy levels of residents, thereby informing targeted measures to enhance health literacy levels. This concerted endeavor is geared toward a paramount objective: elevating the overall health literacy of the nation’s population, thereby charting a course toward comprehensive wellness and informed health care engagement.

In conclusion, the use of machine learning and nomograms for health literacy prediction can deepen the understanding of population health literacy and enable both individuals and policymakers to take proactive measures to improve public health and optimize resource allocation.

### Limitations

This study has several limitations. First, this study used cross-sectional data. Although our data showed correlations between variables, due to the nature of the data and the study design, we were unable to determine if these relationships were causal. Future studies may need to use a longitudinal research design or experimental methods to further explore the relationships between these variables. Second, although the sample size of this study was large enough, at 56,340 residents, the sample was concentrated in Zhejiang, China. The generalizability of the results may still be influenced by regional characteristics; this limits the model’s direct applicability to other provinces or cultural contexts. Future studies could use the same models to validate the findings in other provinces. Finally, as aforementioned, due to the limitations of data accessibility, this study included only basic information about the population in the model and did not include influencing factors such as dietary habits and the availability and utilization of health services. We included social factors such as education, occupation, and income to enhance the model’s accuracy. However, due to the limitations of the research method, it was impossible to capture dynamic changes in economic factors such as income. In the future, the design method can be changed, such as conducting a cohort study and incorporating more comprehensive data, to make the model’s predictions more accurate.

### Conclusion

This study used machine learning and LR analysis models to successfully develop and validate a predictive model for assessing the health literacy level of Chinese adults. The models identified education level, age, annual household income, smoking, and occupation as the most influential determinants of health literacy. This predictive framework provides a practical tool for targeted intervention measures to enhance the health literacy of the population and can guide resource allocation and the formulation of targeted health promotion strategies.

## Supplementary material

10.2196/77654 Multimedia Appendix 1 Test results for the 4 machine learning models, validation results for the random forest model, external validation for nomogram models 1 and 2, and demographic characteristics and average health literacy scores of the cross-sectional study.

## Data Availability

The raw data supporting the conclusions of this article will be made available by the authors, without undue reservation.

## Abbreviations

AUC: area under the curve
DCA: decision curve analysis
FN: false negative
FP: false positive
GBDT: gradient boosting decision tree
HL: health literacy
HL: health literacy
LR: logistic regression
NPV: negative predictive value
OR: odds ratio
PPV: positive predictive value
RF: random forest
ROC: receiver operating characteristic
SHAP: Shapley Additive Explanations
SMOTE: synthetic minority over-sampling technique
TN: true negative
TP: true positive
XGBoost: extreme gradient boosting

## References

[1] Lawless J, Toronto CE, Grammatica GL (2016). Health literacy and information literacy: a concept comparison. RSR.

[2] Simonds SK (1974). Health education as social policy. Health Educ Monogr.

[3] Vries H, Kremers SPJ, Lippke S, Fisher EB, Cameron LD, Christensen AJ, Ehlert U, Guo Y, Oldenburg B (2018). Principles and Concepts of Behavioral Medicine: A Global Handbook.

[4] Berkman ND, Sheridan SL, Donahue KE, Halpern DJ, Crotty K (2011). Low health literacy and health outcomes: an updated systematic review. Ann Intern Med.

[5] Peterson PN, Shetterly SM, Clarke CL (2011). Health literacy and outcomes among patients with heart failure. JAMA.

[6] Devraj R, Gordon EJ (2009). Health literacy and kidney disease: toward a new line of research. Am J Kidney Dis.

[7] Hermans L, Van den Broucke S, Gisle L, Demarest S, Charafeddine R (2021). Mental health, compliance with measures and health prospects during the COVID-19 epidemic: the role of health literacy. BMC Public Health.

[8] Liu Y, Meng H, Tu N, Liu D (2020). The relationship between health literacy, social support, depression, and frailty among community-dwelling older patients with hypertension and diabetes in China. Front Public Health.

[9] Eronen J, Paakkari L, Portegijs E, Saajanaho M, Rantanen T (2021). Health literacy supports active aging. Prev Med.

[10] Kim SH (2009). Health literacy and functional health status in Korean older adults. J Clin Nurs.

[11] National Health Commission (2023). The national health literacy level will reach 27.78% in 2022. China Government Network.

[12] NIH (2024). In 2023, the health literacy level of residents nationwide will increase to 29.70%. China Government Network.

[13] Liu YB, Liu L, Li YF, Chen YL (2015). Relationship between health literacy, health-related behaviors and health status: a survey of elderly Chinese. Int J Environ Res Public Health.

[14] Shi J, Qi L, Li Y, Liu X (2020). Investigation of health literacy status in Beijing, China. Health Lit Res Pract.

[15] Wahl AK, Hermansen Å, Osborne RH, Larsen MH (2021). A validation study of the Norwegian version of the Health Literacy Questionnaire: a robust nine-dimension factor model. Scand J Public Health.

[16] Simpson RM, Knowles E, O’Cathain A (2020). Health literacy levels of British adults: a cross-sectional survey using two domains of the Health Literacy Questionnaire (HLQ). BMC Public Health.

[17] Hæsum LKE, Cichosz SL, Hejlesen OK (2023). Using machine learning to design a short test from a full-length test of functional health literacy in adults-the development of a short form of the Danish TOFHLA. PLoS ONE.

[18] İnceoğlu F, Deniz S, Yagin FH (2023). Prediction of effective sociodemographic variables in modeling health literacy: a machine learning approach. Int J Med Inform.

[19] Zhou R, Yin W, Li W (2022). Prediction model for infectious disease health literacy based on synthetic minority oversampling technique algorithm. Comput Math Methods Med.

[20] Mao N, Wang Y, Zhou A (2026). Mental health literacy predicts depression in older adults in China: an interpretable machine learning model. J Gerontol B Psychol Sci Soc Sci.

[21] Gondo T, Hamada R (2009). Nomogram as predictive model in clinical practice. Gan To Kagaku Ryoho.

[22] Park SY (2018). Nomogram: an analogue tool to deliver digital knowledge. J Thorac Cardiovasc Surg.

[23] Wang X, Lu J, Song Z, Zhou Y, Liu T, Zhang D (2022). From past to future: bibliometric analysis of global research productivity on nomogram (2000–2021). Front Public Health.

[24] Balouktsis A, Chassapis D, Karapantsios TD (2002). A nomogram method for estimating the energy produced by wind turbine generators. Solar Energy.

[25] Vanelslande A, Rousseau P, Lal R, Ghuman BS (1984). Testing the applicability of a soil erodibility nomogram for some tropical soils. Challenges in African Hydrology and Water Resources.

[26] Li Z, Tian Y, Gong Z, Qian L (2021). Health literacy and regional heterogeneities in China: a population-based study. Front Public Health.

[27] Zhang X, Zhang X, Zhang S (2024). The association between health literacy and e-cigarette use: evidence from Zhejiang, China. Front Public Health.

[28] Shen M, Hu M, Liu S, Chang Y, Sun Z (2015). Assessment of the Chinese Resident Health Literacy Scale in a population-based sample in South China. BMC Public Health.

[29] Rong H, Cheng X, Garcia JM (2017). Survey of health literacy level and related influencing factors in military college students in Chongqing, China: a cross-sectional analysis. PLOS ONE.

[30] Chen C, Xu T, Chen Y (2022). Does health literacy promote COVID-19 awareness? Evidence from Zhejiang, China. Front Public Health.

[31] Luo S, Chen W, Hu W (2025). Parental education, own education, and cognitive function in middle-aged and older adults. JAMA Netw Open.

[32] Siddiq H, Maharlouei N, Najand B, Rahmani A, Zare H (2023). Immigration status, educational level, and perceived discrimination in Europe. Int J Environ Res Public Health.

[33] Nie X, Li Y, Li C, Wu J, Li L (2021). The association between health literacy and self-rated health among residents of China aged 15-69 years. Am J Prev Med.

[34] Rafferty AP, Luo H, Winterbauer NL, Bell RA, Little NRG, Imai S (2022). Health literacy among adults with multiple chronic health conditions. J Public Health Manag Pract.

[35] Clouston SAP, Manganello JA, Richards M (2017). A life course approach to health literacy: the role of gender, educational attainment and lifetime cognitive capability. Age Ageing.

[36] Levy H, Janke A (2016). Health literacy and access to care. J Health Commun.

[37] Sagi O, Rokach L (2021). Approximating XGBoost with an interpretable decision tree. Inf Sci (Ny).

[38] An Q, Rahman S, Zhou J, Kang JJ (2023). A comprehensive review on machine learning in healthcare industry: classification, restrictions, opportunities and challenges. Sensors (Basel).

[39] Jose C, Gopakumar G (2019). An improved random forest algorithm for classification in an imbalanced dataset. https://doi.org/10.23919/URSIAP-RASC.2019.8738232.

[40] Liu W, Fan H, Xia M (2022). Credit scoring based on tree-enhanced gradient boosting decision trees. Expert Syst Appl.

[41] Ke G, Meng Q, Finley T, Wang T, Chen W, Ma W (2017). LightGBM: a highly efficient gradient boosting decision tree. Adv Neural Inf Process Syst.

[42] Chen T, Guestrin C (2016). XGBoost: a scalable tree boosting system.

[43] Brownlee J (2016). A gentle introduction to XGBoost for applied machine learning. Machine Learning Mastery.

[44] Chi Z, Liu L, Yi L, Shi L (2025). Prediction of cardiovascular diseases based on GBDT+LR. Sci Rep.

[45] Ren Q, Cheng H, Han H Research on machine learning framework based on random forest algorithm.

[46] Ullah F, Chen X, Rajab K (2022). An efficient machine learning model based on improved features selections for early and accurate heart disease predication. Comput Intell Neurosci.

[47] Gebreyesus Y, Dalton D, Nixon S, De Chiara D, Chinnici M (2023). Machine learning for data center optimizations: feature selection using Shapley Additive exPlanation (SHAP). Future Internet.

[48] He Y, Luo L, Shan R (2023). Development and validation of a nomogram for predicting postoperative early relapse and survival in hepatocellular carcinoma. J Natl Compr Canc Netw.

[49] Van Belle V, Van Calster B (2015). Visualizing risk prediction models. PLOS ONE.

[50] Balachandran VP, Gonen M, Smith JJ, DeMatteo RP (2015). Nomograms in oncology: more than meets the eye. Lancet Oncol.

[51] Moosavi SM, Ghassabian S (2018). Calibration and Validation of Analytical Methods - A Sampling of Current Approaches.

[52] Keam B, Im SA, Park S (2011). Nomogram predicting clinical outcomes in breast cancer patients treated with neoadjuvant chemotherapy. J Cancer Res Clin Oncol.

[53] Lei J, Zhai J, Zhang Y, Qi J, Sun C (2025). Supervised machine learning models for predicting sepsis-associated liver injury in patients with sepsis: development and validation study based on a multicenter cohort study. J Med Internet Res.

[54] Leme D da C, de Oliveira C (2023). Machine learning models to predict future frailty in community-dwelling middle-aged and older adults: the ELSA Cohort Study. J Gerontol A Biol Sci Med Sci.

[55] Thölke P, Mantilla-Ramos YJ, Abdelhedi H (2023). Class imbalance should not throw you off balance: choosing the right classifiers and performance metrics for brain decoding with imbalanced data. Neuroimage.

[56] Xie W, Li Y, Meng X, Zhao M (2023). Machine learning prediction models and nomogram to predict the risk of in-hospital death for severe DKA: a clinical study based on MIMIC-IV, eICU databases, and a college hospital ICU. Int J Med Inform.

[57] Dong W, Xiong S, Lei P (2022). Application of a combined radiomics nomogram based on CE-CT in the preoperative prediction of thymomas risk categorization. Front Oncol.

[58] Xie T, Zhang N, Mao Y, Zhu B (2022). How to predict the electronic health literacy of Chinese primary and secondary school students?: establishment of a model and web nomograms. BMC Public Health.

[59] Li X, Wang Y, Xu J (2022). Development of a machine learning-based risk prediction model for cerebral infarction and comparison with nomogram model. J Affect Disord.

[60] Al Sayah F, Majumdar SR, Egede LE, Johnson JA (2014). Measurement properties and comparative performance of health literacy screening questions in a predominantly low income African American population with diabetes. Patient Educ Couns.

[61] Bonaccorsi G, Zanobini P, Biagi C (2025). Measuring health literacy among seniors: validation of the European Health Literacy Survey Questionnaire 6 in the Tuscan PASSI sample. Ann Ig.

[62] (2023). Introducing the three-year campaign for information exchange and sharing among medical institutions nationwide. National Health Commission of the People’s Republic of China.

[63] Martin LT, Ruder T, Escarce JJ (2009). Developing predictive models of health literacy. J Gen Intern Med.

[64] Long Y, Jia C, Luo X (2022). The impact of higher education on health literacy: a comparative study between urban and rural China. Sustainability.

[65] Bujnowska-Fedak M, Grata-Borkowska U (2015). Use of telemedicine-based care for the aging and elderly: promises and pitfalls. SHTT.

[66] Riiser K, Helseth S, Haraldstad K, Torbjørnsen A, Richardsen KR (2020). Adolescents’ health literacy, health protective measures, and health-related quality of life during the Covid-19 pandemic. PLOS ONE.

[67] Mullan J, Burns P, Weston K (2017). Health literacy amongst health professional university students: a study using the Health Literacy Questionnaire. Education Sciences.

[68] Eisapareh K, Nazari M, Kaveh MH, Ghahremani L (2022). The relationship between job stress and health literacy with the quality of work life among Iranian industrial workers: the moderating role of social support. Curr Psychol.

[69] Svendsen MT, Bak CK, Sørensen K (2020). Associations of health literacy with socioeconomic position, health risk behavior, and health status: a large national population-based survey among Danish adults. BMC Public Health.

[70] Furuya Y, Kondo N, Yamagata Z, Hashimoto H (2015). Health literacy, socioeconomic status and self-rated health in Japan. Health Promot Int.

[71] Shayakhmetov SS, Toguzbayeva KK, Ismailova AA, Tabibi R, Derbishalieva ZK, Dzhusupov KO (2020). Health literacy of rural population of Kazakhstan. ijph.

[72] Marques SRL, Escarce AG, Lemos SMA (2018). Health literacy and self-rated health in adults primary care patients. Codas.

[73] Nielsen-Bohlman L, Panzer AM, Kindig DA (2004). Health Literacy: A Prescription to End Confusion.

[74] Basirimoghadam M, Rafii F, Ebadi A (2020). Self-rated health and general procrastination in nurses: a cross-sectional study. Pan Afr Med J.

[75] Odoh C, Vidrine JI, Businelle MS, Kendzor DE, Agrawal P, Reitzel LR (2019). Health literacy and self-rated health among homeless adults. Health Behav Res.

[76] Azlan AA, Hamzah MR, Tham JS, Ayub SH, Ahmad AL, Mohamad E (2021). Associations between health literacy and sociodemographic factors: a cross-sectional study in Malaysia utilising the HLS-M-Q18. Int J Environ Res Public Health.

[77] Tzenios N (2019). The impact of health literacy on employee productivity: an empirical investigation. Empir Quests Manag Essences.

[78] Ed-daoudy A, Maalmi K (2019). A new Internet of Things architecture for real-time prediction of various diseases using machine learning on big data environment. J Big Data.

