# Supplementary material for: Interpretable Machine Learning–Derived Nomogram Model to Predict Population Health Literacy: Cross-Sectional Development and Validation Study
Source: JMIR Med Inform. 2026 Oct 13;14:e77654. doi: 10.2196/77654 (PMC13660971; doi:10.2196/77654)
Supplement: Supplementary file 1 [file medinform-v14-e77654-s001.docx]

**Table S1. The four machine learning models (GBDT, XGBoost, LR and RF) constructed based on the 2023 data were tested at the 0.1-0.9 threshold for various performance indicators of the validation set.**

| **Model** | **AUC** | **Thresholds** | **TN/TP/FN/FP** | **Sensitivity** | **Specificity** | **Accuracy** | **Balanced accuracy** | **PPV** | **NPV** |
| --- | --- | --- | --- | --- | --- | --- | --- | --- | --- |
| GBDT | 0.807  (0.798,0.816) | 0.1 | 2167/3496/147/5458 | 0.960  (0.953,0.966) | 0.284  (0.274,0.294) | 0.503  (0.493,0.512) | 0.622  (0.616,0.628) | 0.390  (0.380,0.401) | 0.936  (0.927,0.946) |
|  |  | 0.2 | 3811/3263/380/3814 | 0.896  (0.886,0.906) | 0.500  (0.489,0.511) | 0.628  (0.619,0.637) | 0.698  (0.690,0.705) | 0.461 (0.449,0.473) | 0.909 (0.901,0.918) |
|  |  | 0.3 | 5005/2951/692/2620 | 0.810  (0.797,0.823) | 0.656  (0.646,0.667) | 0.706 (0.698,0.714) | 0.733 (0.725,0.742) | 0.530  (0.517,0.543) | 0.879 (0.870,0.887) |
|  |  | 0.4 | 5775/2606/1037/1850 | 0.715  (0.701,0.730) | 0.757 (0.748,0.767) | 0.744 (0.736,0.752) | 0.736 (0.728,0.745) | 0.585 (0.570,0.599) | 0.848 (0.839,0.856) |
|  |  | 0.5 | 6248/2310/1333/1377 | 0.634 (0.618,0.650) | 0.819 (0.811,0.828) | 0.759 (0.752,0.767) | 0.727 (0.718,0.736) | 0.627 (0.611,0.642) | 0.824 (0.816,0.833) |
|  |  | 0.6 | 6618/1955/1688/1007 | 0.537  (0.520,0.553) | 0.868 (0.860,0.876) | 0.761 (0.753,0.769) | 0.702 (0.693,0.711) | 0.660 (0.643,0.677) | 0.797 (0.788,0.805) |
|  |  | 0.7 | 7031/1521/2122/594 | 0.418  (0.401,0.434) | 0.922 (0.916,0.928) | 0.759  (0.751,0.767) | 0.670 (0.661,0.678) | 0.719 (0.700,0.738) | 0.768 (0.760,0.777) |
|  |  | 0.8 | 7421/713/2930/204 | 0.196 (0.183,0.209) | 0.973 (0.970,0.977) | 0.722  (0.714,0.730) | 0.584 (0.578,0.591) | 0.778 (0.751,0.804) | 0.717 (0.708,0.726) |
|  |  | 0.9 | 7624/1/3642/1 | 0.000 | 1.000 | 0.677 (0.668,0.685) | 0.500 | 0.500 (0.000,1.000) | 0.677 (0.668,0.685) |
| XGBoost | 0.789 (0.780,0.799) | 0.1 | 2697/3413/230/4928 | 0.937  (0.929,0.945) | 0.354 (0.343,0.364) | 0.542 (0.533,0.551) | 0.645  (0.639,0.652) | 0.409 (0.399,0.420) | 0.921 (0.912,0.931) |
|  |  | 0.2 | 4001/3143/500/3624 | 0.863  (0.852,0.874) | 0.525 (0.514,0.536) | 0.634 (0.625,0.643) | 0.694 (0.686,0.702) | 0.464  (0.453,0.476) | 0.889 (0.880,0.898) |
|  |  | 0.3 | 4967/2850/793/2658 | 0.782 (0.769,0.796) | 0.651 (0.641,0.662) | 0.694 (0.685,0.702) | 0.717  (0.708,0.725) | 0.517 (0.504,0.531) | 0.862 (0.853,0.871) |
|  |  | 0.4 | 5612/2598/1045/2013 | 0.713 (0.698,0.728) | 0.736 (0.726,0.746) | 0.729 (0.720,0.737) | 0.725 (0.716,0.733) | 0.563 (0.549,0.578) | 0.843  (0.834,0.852) |
|  |  | 0.5 | 6133/2291/1352/1492 | 0.629  (0.613,0.645) | 0.804 (0.795,0.813) | 0.748 (0.740,0.756) | 0.717 (0.708,0.726) | 0.606 (0.590,0.621) | 0.819 (0.811,0.828) |
|  |  | 0.6 | 6505/1974/1669/1120 | 0.542 (0.526,0.558) | 0.853 (0.845,0.861) | 0.752 (0.745,0.760) | 0.697 (0.688,0.707) | 0.638 (0.621,0.655) | 0.796 (0.787,0.805) |
|  |  | 0.7 | 6852/1620/2023/773 | 0.445 (0.429,0.461) | 0.899 (0.892,0.905) | 0.752 (0.744,0.760) | 0.672 (0.663,0.680) | 0.677 (0.658,0.696) | 0.772 (0.763,0.781) |
|  |  | 0.8 | 7181/1148/2495/444 | 0.315 (0.300,0.330) | 0.942 (0.937,0.947) | 0.739 (0.731,0.747) | 0.628 (0.620,0.636) | 0.721 (0.699,0.743) | 0.742 (0.733,0.751) |
|  |  | 0.9 | 7487/420/3223/138 | 0.115 (0.105,0.126) | 0.982 (0.979,0.985) | 0.702 (0.693,0.710) | 0.549 (0.543,0.554) | 0.753 (0.717,0.788) | 0.699 (0.690,0.708) |
| LR | 0.809 (0.799,0.818) | 0.1 | 1100/3596/47/6525 | 0.987 (0.983,0.991) | 0.144 (0.136,0.152) | 0.417  (0.408,0.426) | 0.566 (0.561,0.570) | 0.355 (0.346,0.365) | 0.959 (0.948,0.970) |
|  |  | 0.2 | 3177/3396/247/4448 | 0.932  (0.924,0.940) | 0.417 (0.406,0.428) | 0.583 (0.574,0.592) | 0.674 (0.668,0.681) | 0.433 (0.422,0.444) | 0.928 (0.919,0.937) |
|  |  | 0.3 | 4512/3086/557/3113 | 0.847 (0.835,0.859) | 0.592 (0.581,0.603) | 0.674 (0.666,0.683) | 0.719 (0.711,0.727) | 0.498 (0.485,0.510) | 0.890 (0.882,0.899) |
|  |  | 0.4 | 5519/2725/918/2106 | 0.748 (0.734,0.762) | 0.724 (0.714,0.734) | 0.732 (0.723,0.740) | 0.736 (0.727,0.745) | 0.564 (0.550,0.578) | 0.857 (0.849,0.866) |
|  |  | 0.5 | 6030/2476/1167/1595 | 0.680 (0.665,0.695) | 0.791 (0.782,0.800) | 0.755  (0.747,0.763) | 0.735  (0.726,0.744) | 0.608 (0.593,0.623) | 0.838 (0.829,0.846) |
|  |  | 0.6 | 6377/2233/1410/1248 | 0.613 (0.597,0.629) | 0.836 (0.828,0.845) | 0.764 (0.756,0.772) | 0.725 (0.716,0.734) | 0.641 (0.626,0.657) | 0.819 (0.810,0.827) |
|  |  | 0.7 | 6747/1871/1772/878 | 0.514 (0.497,0.530) | 0.885 (0.878,0.892) | 0.765  (0.757,0.773) | 0.699 (0.690,0.708) | 0.681 (0.663,0.698) | 0.792 (0.783,0.801) |
|  |  | 0.8 | 7142/1298/2345/483 | 0.356 (0.341,0.372) | 0.937 (0.931,0.942) | 0.749 (0.741,0.757) | 0.646 (0.638,0.655) | 0.729 (0.708,0.749) | 0.753 (0.744,0.762) |
|  |  | 0.9 | 7617/16/3627/8 | 0.004 (0.002,0.007) | 0.999  (0.998,1.000) | 0.677 (0.669,0.686) | 0.502 (0.501,503) | 0.667 (0.478,0.855) | 0.677 (0.669,0.686) |
| RF | 0.813 (0.804,0.822) | 0.1 | 828/3609/34/6797 | 0.991  (0.988,0.994) | 0.109  (0.102,0.116) | 0.394 (0.385,0.403) | 0.550 (0.546,0.553) | 0.347 (0.338,0.356) | 0.961  (0.948,0.974) |
|  |  | 0.2 | 3081/3424/219/4544 | 0.940 (0.932,0.948) | 0.404 (0.393,0.415) | 0.577 (0.568,0.586) | 0.672 (0.665,0.679) | 0.430 (0.419,0.441) | 0.934 (0.925,0.942) |
|  |  | 0.3 | 4516/3103/540/3109 | 0.852 (0.840,0.863) | 0.592 (0.581,0.603) | 0.676 (0.668,0.685) | 0.722 (0.714,0.730) | 0.500  (0.487,0.512) | 0.893 (0.885,0.902) |
|  |  | 0.4 | 5444/2822/821/2181 | 0.775 (0.761,0.788) | 0.714 (0.704,0.724) | 0.734 (0.725,0.742) | 0.744 (0.736,0.753) | 0.564 (0.550,0.578) | 0.869 (0.861,0.877) |
|  |  | 0.5 | 6063/2475/1168/1562 | 0.679 (0.664,0.695) | 0.795 (0.786,0.804) | 0.758 (0.750,0.766) | 0.737 (0.728,0.746) | 0.613 (0.598,0.628) | 0.838 (0.830,0.847) |
|  |  | 0.6 | 6394/2211/1432/1231 | 0.607 (0.591,0.623) | 0.839  (0.830,0.847) | 0.764 (0.756,0.772) | 0.723  (0.714,0.732) | 0.642 (0.626,0.658) | 0.817 (0.808,0.826) |
|  |  | 0.7 | 6865/1735/1908/760 | 0.476 (0.460,0.492) | 0.900 (0.894,0.907) | 0.763  (0.755,0.771) | 0.688  (0.680,0.697) | 0.695 (0.677,0.713) | 0.783  (0.774,0.791) |
|  |  | 0.8 | 7307/1015/2628/318 | 0.279 (0.264,0.293) | 0.958 (0.954,0.963) | 0.739  (0.730,0.747) | 0.618 (0.611,0.626) | 0.761 (0.739,0.784) | 0.735  (0.727,0.744) |
|  |  | 0.9 | 7625/0/3643/0 | 0.000 | 1.000 | 0.677 (0.668,0.685) | 0.500 | 0.000 | 0.677 (0.668,0.685) |

Note: TP: True Positive, TN: True Negative, FP: False Positive, FN: False Negative

**Table S2. External validation of the random forest model (developed based on the 2023 data) on the 2024 dataset, as well as the performance indicators under the threshold range of 0.1 to 0.9.**

| **Model** | **AUC** | **Threshold** | **TN/TP/FN/FP** | **Sensitivity** | **Specificity** | **Accuracy** | **Balanced Accuracy** | **PPV** | **NPV** |
| --- | --- | --- | --- | --- | --- | --- | --- | --- | --- |
| RF | 0.815  (0.811,0.819) | 0.1 | 4179/19066/202/33416 | 0.990 (0.988,0.991) | 0.111 (0.108,0.114) | 0.409 (0.405,0.413) | 0.550 (0.549,0.552) | 0.363 (0.359,0.367) | 0.954 (0.948,0.960) |
|  |  | 0.2 | 15210/18082/1186/22385 | 0.938 (0.935,0.942) | 0.405 (0.400,0.410) | 0.585 (0.581,0.590) | 0.672 (0.669,0.675) | 0.447  (0.442,0.452) | 0.928 (0.924,0.932) |
|  |  | 0.3 | 22943/16400/2868/14652 | 0.851 (0.846,0.856) | 0.610 (0.605,0.615) | 0.692 (0.688,0.696) | 0.731 (0.727,0.734) | 0.528  (0.523,0.534) | 0.889  (0.885,0.893) |
|  |  | 0.4 | 27307/14780/4488/10288 | 0.767 (0.761,0.773) | 0.726 (0.722,0.731) | 0.740 (0.737,0.744) | 0.747 (0.743,0.750) | 0.590  (0.584,0.596) | 0.859  (0.855,0.863) |
|  |  | 0.5 | 30193/13128/6140/7402 | 0.681 (0.675,0.688) | 0.803 (0.799,0.807) | 0.762 (0.758,0.765) | 0.742 (0.738,0.746) | 0.639  (0.633,0.646) | 0.831  (0.827,0.835) |
|  |  | 0.6 | 31805/11644/7624/5790 | 0.604 (0.597,0.611) | 0.846  (0.842,0.850) | 0.764 (0.761,0.768) | 0.725 (0.721,0.729) | 0.668  (0.661,0.675) | 0.807 (0.803,0.811) |
|  |  | 0.7 | 34064/8924/10344/3531 | 0.463 (0.456,0.470) | 0.906 (0.903,0.909) | 0.756 (0.752,0.760) | 0.685 (0.681,0.688) | 0.716  (0.709,0.724) | 0.767 (0.763,0.771) |
|  |  | 0.8 | 36056/5068/14200/1539 | 0.263 (0.257,0.269) | 0.959 (0.957,0.961) | 0.723 (0.720,0.727) | 0.611 (0.608,0.614) | 0.767  (0.757,0.777) | 0.717 (0.714,0.721) |
|  |  | 0.9 | 37595/0/19268/0 | 0.000 | 1.000 | 0.661 (0.657,0.665) | 0.500 |  | 0.661 (0.657,0.665) |

Note: TP: True Positive, TN: True Negative, FP: False Positive, FN: False Negative

**Table S3. The external validation of the nomogram models 1 and 2 (developed based on 2023 data) on the 2024 dataset and the performance indicators under the threshold of 0.1 - 0.9.**

| **Model** | **AUC** | **Thresholds** | **TN/TP/FN/FP** | **Sensitivity** | **Specificity** | **Accuracy** | **Balanced accuracy** | **PPV** | **NPV** |
| --- | --- | --- | --- | --- | --- | --- | --- | --- | --- |
| Model 1 | 0.814 (0.810,0.817) | 0.1 | 12251/18451/817/25344 | 0.958  (0.955,0.960) | 0.326 (0.321,0.331) | 0.540 (0.536,0.544) | 0.642 (0.639,0.644) | 0.421  (0.417,0.426) | 0.937  (0.933,0.942) |
|  |  | 0.2 | 22759/16419/2849/14836 | 0.852 (0.847,0.857) | 0.605 (0.600,0.610) | 0.689 (0.685,0.693) | 0.729 (0.725,0.732) | 0.525  (0.520,0.531) | 0.889 (0.885,0.893) |
|  |  | 0.3 | 28455/14122/5146/9140 | 0.733 (0.727,0.739) | 0.757  (0.753,0.761) | 0.749 (0.745,0.752) | 0.745 (0.741,0.749) | 0.607 (0.601,0.613) | 0.847 (0.843,0.851) |
|  |  | 0.4 | 30712/12608/6660/6883 | 0.654  (0.648,0.661) | 0.817 (0.813,0.821) | 0.762 (0.758,0.765) | 0.736 (0.732,0.740) | 0.647 (0.640,0.654) | 0.822 (0.818,0.826) |
|  |  | 0.5 | 32364/10959/8309/5231 | 0.569 (0.562,0.576) | 0.861 (0.857,0.864) | 0.762 (0.758,0.765) | 0.715 (0.711,0.719) | 0.677 (0.670,0.684) | 0.796 (0.792,0.800) |
|  |  | 0.6 | 33976/9117/10151/3619 | 0.473 (0.466,0.480) | 0.904 (0.901,0.907) | 0.758 (0.754,0.761) | 0.688  (0.685,0.692) | 0.716 (0.708,0.724) | 0.770 (0.766,0.774) |
|  |  | 0.7 | 35668/5864/13404/1927 | 0.304  (0.298,0.311) | 0.949  (0.946,0.951) | 0.731 (0.727,0.734) | 0.627 (0.623,0.630) | 0.753 (0.743,0.762) | 0.727  (0.723,0.731) |
|  |  | 0.8 | 37454/560/18708/141 | 0.029 (0.027,0.032) | 0.996 (0.996,0.997) | 0.669 (0.665,0.672) | 0.513 (0.511,0.514) | 0.799 (0.768,0.827) | 0.667 (0.663,0.671) |
|  |  | 0.9 | 37595/0/19268/0 | 0.000 | 1.000 | 0.661 (0.657,0.665) | 0.500 |  | 0.661 (0.657,0.665) |
| Model 2 | 0.812  (0.808,0.815) | 0.1 | 12116/18461/807/25479 | 0.958 (0.955,0.961) | 0.322 (0.318,0.327) | 0.538 (0.534,0.542) | 0.640 (0.637,0.643) | 0.420 (0.416,0.425) | 0.938 (0.933,0.942) |
|  |  | 0.2 | 22940/16350/2918/24655 | 0.849 (0.843,0.854) | 0.610 (0.605,0.615) | 0.691 (0.687,0.695) | 0.729 (0.726,0.733) | 0.527 (0.522,0.533) | 0.887 (0.883,0.891) |
|  |  | 0.3 | 28333/14129/5139/9262 | 0.733  (0.727,0.739) | 0.754 (0.749,0.758) | 0.747 (0.743,0.750) | 0.743 (0.740,0.747) | 0.604 (0.598,0.610) | 0.846 (0.843,0.850) |
|  |  | 0.4 | 30696/12582/6686/6899 | 0.653 (0.646,0.660) | 0.816 (0.813,0.820) | 0.761 (0.758,0.765) | 0.735 (0.731,0.739) | 0.646 (0.639,0.653) | 0.821 (0.817,0.825) |
|  |  | 0.5 | 32344/10855/8413/5251 | 0.563 (0.556,0.570) | 0.860  (0.857,0.864) | 0.760 (0.756,0.763) | 0.712 (0.708,0.716) | 0.674 (0.667,0.681) | 0.794 (0.790,0.797) |
|  |  | 0.6 | 33892/9187/10081/3703 | 0.477 (0.470,0.484) | 0.902 (0.898,0.904) | 0.758 (0.754,0.761) | 0.689 (0.685,0.693) | 0.713  (0.705,0.721) | 0.771 (0.767,0.775) |
|  |  | 0.7 | 35542/5962/13306/2053 | 0.309 (0.303,0.316) | 0.945 (0.943,0.948) | 0.730 (0.726,0.734) | 0.627 (0.624,0.631) | 0.744 (0.734,0.753) | 0.728 (0.724,0.732) |
|  |  | 0.8 | 37466/408/18860/129 | 0.021 (0.019,0.023) | 0.997 (0.996,0.997) | 0.666 (0.662,0.670) | 0.509 (0.508,0.510) | 0.760 (0.722,0.794) | 0.665 (0.661,0.669) |
|  |  | 0.9 | 37595/0/19268/0 | 0.000 | 1.000 | 0.661 (0.657,0.665) | 0.500 |  | 0.661 (0.657,0.665) |

Note: TP: True Positive, TN: True Negative, FP: False Positive, FN: False Negative

**Table S4. The demographic characteristics and average health literacy scores of the research population in the 2024 cross-sectional study in Zhejiang Province.**

| Predictors | | All N (%) | Health Literacy  Score ($\bar{\text{X}}\text{±}\text{SD}$) |
| --- | --- | --- | --- |
| Overall | | 56863 (100%) | 40.906$\text{±}$12.347 |
| Age | 15-33 | 7873 (13.85%) | 52.821$\text{±}$8.353 |
|  | 34-41 | 7335 (12.90%) | 51.620$\text{±}$9.358 |
|  | 42-49 | 8390 (14.75%) | 48.770$\text{±}$11.192 |
|  | 50-54 | 7225 (12.71%) | 44.220$\text{±}$12.721 |
|  | 55-59 | 8902 (15.66%) | 40.419$\text{±}$13.046 |
|  | 60-64 | 9008 (15.84%) | 38.514$\text{±}$13.019 |
|  | 65-69 | 8130 (14.30%) | 35.608$\text{±}$13.112 |
| Gender | Male | 26376 (46.39%) | 41.214$\text{±}$12.184 |
|  | Female | 30487 (53.61%) | 40.640$\text{±}$12.481 |
| Ethnicity | Han | 56000 (98.48%) | 40.914$\text{±}$12.338 |
|  | Other | 863 (1.52%) | 40.340$\text{±}$12.915 |
| Educational level | Primary school and below | 15170 (26.68%) | 31.70$\text{±}$12.04 |
|  | Junior high school | 17987 (31.63%) | 39.22$\text{±}$11.28 |
|  | Senior high school | 10049 (17.67%) | 45.32$\text{±}$9.30 |
|  | Higher education | 13657 (24.02%) | 50.10$\text{±}$6.78 |
| Marital status | Single/Widow/Divorced | 10614 (18.67%) | 42.841$\text{±}$12.105 |
|  | Married | 46249 (81.33%) | 40.462$\text{±}$12.360 |
| Occupation | Technical/Professional(Occupation_1) | 6751 (11.87%) | 48.958$\text{±}$8.514 |
|  | Student(Occupation_2) | 1701 (2.99%) | 48.216$\text{±}$7.616 |
|  | Manual(Occupation_3) | 27316 (48.04%) | 36.539$\text{±}$12.517 |
|  | Commercial(Occupation_4) | 12480 (21.95%) | 45.648$\text{±}$10.057 |
|  | Unemployed/Others | 8615 (15.15%) | 40.129$\text{±}$11.823 |
| Family members | 1-2 | 18582 (32.68%) | 38.459$\text{±}$12.736 |
|  | 3-5 | 32644 (57.41%) | 42.426$\text{±}$11.865 |
|  | >5 | 5637 (9.91%) | 40.168$\text{±}$12.422 |
| Annual household income | 0-49,999 | 14859 (26.13%) | 35.728$\text{±}$12.971 |
|  | 50,000-99,999 | 13833 (24.33%) | 39.250$\text{±}$12.102 |
|  | 100,000-149,999 | 13106 (23.05%) | 42.674$\text{±}$11.352 |
|  | ≥150,000 | 15065 (26.49%) | 45.995$\text{±}$10.484 |
| Local registered residence | Yes | 52776 (92.81%) | 40.793$\text{±}$12.407 |
|  | No | 4087 (7.19%) | 42.370$\text{±}$11.441 |
| Smoking | No | 46670 (82.07%) | 41.211$\text{±}$12.319 |
|  | Yes | 10193 (17.93%) | 39.508$\text{±}$12.380 |
| Chronic disease | No | 39925 (70.21%) | 42.452$\text{±}$11.935 |
|  | Yes | 16938 (29.79%) | 37.262$\text{±}$12.534 |
| Self-rated health | Very Good | 18657 (32.81%) | 41.319$\text{±}$12.386 |
|  | Good | 19238 (33.83%) | 43.063$\text{±}$11.573 |
|  | Fair | 16683 (29.34%) | 38.822$\text{±}$12.473 |
|  | Bad | 1824 (3.21%) | 35.641$\text{±}$12.569 |
|  | Very Bad | 461 (0.81%) | 30.416$\text{±}$13.264 |
